# Differential Operant Conditioning of Emotional‐Motivational and Sensory‐Discriminative Pain Responses

**DOI:** 10.1002/ejp.70162

**Published:** 2025-11-19

**Authors:** Melissa L. Flury, Martin Löffler, Shaili Gour, Susanne Becker

**Affiliations:** ^1^ Integrative Spinal Research Group, Department of Chiropractic Medicine, Balgrist University Hospital Zurich University of Zurich Zurich Switzerland; ^2^ University of Zurich Zurich Switzerland; ^3^ Neuroscience Center Zurich University of Zurich and ETH Zurich Zurich Switzerland; ^4^ Institute of Cognitive and Clinical Neuroscience, Central Institute of Mental Health, Medical Faculty Mannheim Heidelberg University Mannheim Germany; ^5^ Clinical Psychology, Department of Experimental Psychology Heinrich Heine University Düsseldorf Germany

## Abstract

**Background:**

The experience of pain consists of different components, including sensory‐discriminative and emotional‐motivational components. While these components are often well aligned, they can also dissociate. Operant conditioning may selectively modulate one component without affecting the other. However, evidence directly comparing operant conditioning effects on both emotional‐motivational and sensory‐discriminative components of pain is lacking. The aim of the present study was to test whether operant conditioning would differentially affect behavioral surrogate measures of emotional‐motivational and sensory‐discriminative pain responses.

**Methods:**

62 healthy participants performed in two testing sessions a pain avoidance task to assess emotional‐motivational pain responses and a temperature discrimination task to assess sensory‐discriminative pain responses (counterbalanced order). In the second half of each task, successful pain avoidance or accurate temperature discrimination was followed by monetary reinforcement.

**Results:**

Contingent reinforcement selectively enhanced pain avoidance, evidenced by faster reaction times and increased success rates, while temperature discrimination performance remained unchanged, likely due to a ceiling effect in task difficulty. Operantly conditioned changes in pain behaviour did not generalize to self‐reported pain intensity and unpleasantness ratings.

**Conclusions:**

These findings indicate a modulation of emotional–motivational pain processing by operant conditioning, while effects on sensory–discriminative processing remain absent. These results support the idea that enhanced pain perception in chronic pain can be induced by operant learning, potentially through the specific learning of increased emotional–motivational pain responses.

**Significance Statement:**

This study demonstrates that operant conditioning can enhance avoidance responses, serving as an indicator of emotional–motivational pain responses. In contrast, sensory–discriminative aspects of pain were not modulated by operant conditioning. These results confirm the important role of operant conditioning specifically in emotional–motivational pain processing. This insight may help explain how learning contributes to increased emotional–motivational pain processing in chronic pain.

## 1 Introduction

Pain is a multidimensional phenomenon (de Souza et al. 2017; Goldberg and McGee 2011). Melzack's initial tripartite model of pain proposes that pain comprises sensory‐discriminative, emotional‐motivational, and cognitive‐evaluative components (Melzack and Casey 1968). Thus, the pain experience is more than the conscious perception of nociceptive signals: While sensory‐discriminative processes refer to quality, intensity and spatiotemporal features of the sensation, emotional‐motivational processes refer to its valence and typically aversiveness, and cognitive‐evaluative processes modulate the previous components, determining together pain responses (Melzack and Casey 1968; Rainville et al. 1999; Vachon‐Presseau et al. 2016). Sensory‐discriminative and emotional‐motivational aspects of pain are typically aligned, but they can also dissociate (Auvray et al. 2010). As everyday examples of such dissociations, many people perceive painful massages as pleasant or enjoy eating hot chilies. Although the literature has so far focused more on different response channels (e.g., behavioural vs. self‐reports) than on different pain components, some studies suggest these components dissociate using operant conditioning (Becker et al. 2008, 2011, 2012; Hölzl et al. 2005). This finding highlights the importance of operant conditioning in pain. Moreover, such learning appears pathogenetically relevant, as it seems to be altered in chronic pain patients (Becker et al. 2011).

In chronic pain, emotional‐motivational components likely dominate over sensory‐discriminative components. The influential fear‐avoidance model of pain referred in its earliest version to chronic pain as “exaggerated pain perception” due to disproportionally augmented emotional‐motivational relative to sensory‐discriminative pain components (Lethem et al. 1983). Such heightened emotional‐motivational pain perception is further mirrored in chronic pain patients' increased pain avoidance behaviours (Gatchel et al. 2016; Lethem et al. 1983; Meulders 2019; Minami 2019; Vlaeyen and Linton 2000). Similarly, previous findings suggest that chronic pain processing is characterised by a functional shift from nociceptive to non‐nociceptive emotional brain circuitry (Hashmi et al. 2013). This shift also aligns with increased negative affect, impaired motivated behaviour, and the inability to feel pleasure (i.e., anhedonia) in chronic pain as well as very high comorbidities (up to 86%) between chronic pain and affective disorders (Poole et al. 2009). Accordingly, chronic pain has been suggested to induce a negative hedonic shift (Borsook et al. 2016). Despite these findings, the mechanisms underlying such increased emotional‐motivational pain processing and this negative hedonic shift have not been investigated so far.

This study aimed to show that emotional‐motivational and sensory‐discriminative pain components can be increased independently via operant conditioning. We hypothesized that contingent reinforcement in the avoidance task enhances avoidance behaviour and increases pain unpleasantness without affecting perceived pain intensity, while contingent reinforcement in the discrimination task improves discrimination performance and increases perceived intensity without altering perceived unpleasantness. The focus was on gaining a mechanistic understanding in healthy states as a needed basis. Therefore, healthy volunteers performed two psychophysical tasks in two experimental sessions: a discrimination task assessing sensory‐discriminative and an avoidance task assessing emotional‐motivational pain components. After baseline assessment of pain avoidance behaviour and pain discrimination, contingent (experimental condition) or noncontingent (control condition) monetary rewards were used to modulate pain avoidance behaviour and pain discrimination.

## 2 Methods

### 2.1 Participants

In total, 62 participants (29 females; age: *M* = 29.98 years, SD = 9.91 years) who did not meet any exclusion criteria were included in the study. An a priori sample size calculation using G*power 3.1 (Faul et al. 2007) for an *F*‐test based on a basic model that includes a within‐subject factor “phase” (baseline vs. learning) and a between‐subject factor “contingency” (contingent vs. noncontingent) for both the discrimination and the avoidance task was performed. The sample size calculation was performed based on the statistical model assessing the main factors of interest. The calculation assumed a desired medium effect size *f* = 0.25, a 5% probability for committing a Type I error (*α* = 0.05), a 20% probability for committing a Type II error (1 − *ß* = 0.20), and an attrition rate of 10%. Based on these parameters, the required sample size was 62 participants. A desired medium effect size was chosen because no comparable experiments have been performed before, and the true effect size is thus unknown.

Participants were excluded if they reported pain on more than 30 days in the 12 months prior to participation, any chronic pain, major medical or psychiatric conditions, or pregnancy. They were asked to abstain from alcohol, drugs, and analgesics 24 h before the experimental sessions that they attended. The study was approved by the local ethics committee and informed consent was obtained from all participants according to the latest revised Declaration of Helsinki (World Medical Association 2013). The study was preregistered at ClinicalTrials.gov (Identifier: NCT04280796; Protocol ID: PR00P1_179697/1).

Four participants were excluded from the statistical analyses: One participant withdrew from the study, and in the other three, major technical errors occurred during data collection. The final sample thus consisted of 58 participants (27 females; age: *M* = 30.03 years, SD = 10.02 years).

### 2.2 Thermal Stimulation

The participants received heat stimuli applied with a 30 mm diameter contact thermode (Contact Heat Evoked Potentials, CHEPS; PATHWAY Pain & Sensory Evaluation System, Medoc Ltd. Advanced Medical System, Israel) on the thenar of their non‐dominant hand. To standardise the thenar stimulation, participants placed their hand on a hemisphere made of Styrofoam in which the thermode was embedded. The baseline thermode temperature was kept constant at 35°C. To achieve comparable percepts of the stimulation across participants as a necessary baseline for interpreting the results, target temperatures were adjusted to the individual pain sensitivity (see section “Pain Assessments”).

To minimise the risk of skin burns, the maximum temperature for the experimental tasks was calculated with the skin burn risk formula [(−0.3*temp_mean) + 15.2]/log100(time_m), with *temp_mean* being the mean temperature applied over a certain amount of time, the latter being given by *time_m* in minutes, allowing an estimation of the potential risk of skin burns, with a critical value smaller than 1 (Becker et al. 2008; Kleinböhl et al. 1999). In addition, the maximum temperature was kept at 50°C.

### 2.3 Rating Scales

Throughout the experimental sessions, participants rated perceived intensity and un‐/pleasantness of some of the applied thermal stimuli using horizontally orientated visual analogue scales (VAS) displayed on a screen in front of them. The VAS for the intensity rating ranged from 0 = “no sensation” to 200 = “most intense pain tolerable” with 100 being the “pain threshold” to differentiate between non‐painful (range between 0 and 99) and painful sensations (range between 101 and 200) (Becker, Gandhi, et al. 2013; Becker et al. 2015). The VAS for the un‐/pleasantness rating ranged from −100 = “extremely unpleasant” to +100 = “extremely pleasant” with 0 being “neutral” to differentiate between unpleasant (negative range between −100 and −1) and pleasant sensations (positive range between +1 and +100) (Becker, Gandhi, et al. 2013; Becker et al. 2015). Participants gave their ratings by moving a slider on the VASs using the right and left arrow keys of a keyboard in front of them.

### 2.4 General Procedure and Study Design

**FIGURE 1:**
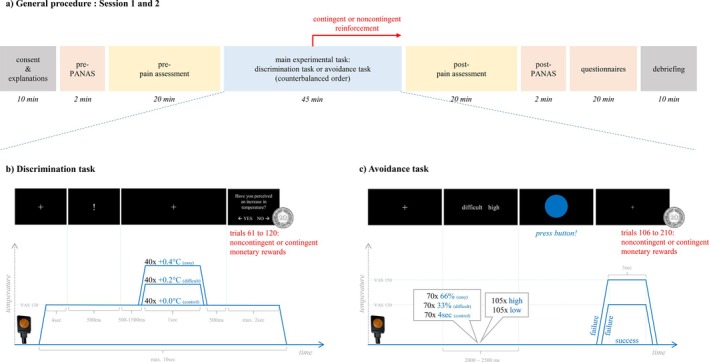
General procedure and study design. The within‐subject study design included two experimental sessions separated ideally exactly 1 week apart. (a) General procedure. Consent & explanations: Written consent was given by the participants, the inclusion and exclusion criteria were checked, and the purpose and course of the study as well as the used material was explained. Pre‐/Post‐PANAS: the positive and negative Affect Schedule (PANAS) was filled out to assess mood. Pre‐pain assessment: (i) assessment of the individual thermal pain threshold and thermal pain tolerance, (ii) visual analogue scale (VAS) ratings of intensity and un‐/pleasantness of two sets of four different heat stimuli in predetermined order, and (iii) determination of the stimulus intensity for the main experimental tasks using VAS ratings of a series of different heat stimuli. Post‐pain assessment: (i) assessment of the individual thermal pain threshold and thermal pain tolerance, and (ii) VAS ratings of the intensity and un‐/pleasantness of two sets of four different heat stimuli in a predetermined order. Main experimental task: the participants perform a discrimination task and an avoidance task in counterbalanced order. In both tasks, monetary reinforcement is implemented after half of the trials. Participants received contingent reinforcement in one of the tasks and noncontingent reinforcement as a control condition in the other task. Questionnaires: completion of questionnaires. Debriefing: exit interview including a brief explanation of the study aims. (b) Discrimination task. The experimental heat stimulus rose from baseline to the pre‐determined individual target temperature. After the presentation of an exclamation mark on the screen, the temperature might further increase by 0.4°C (easy condition), by 0.2°C (difficult condition), or it might remain at the target temperature (+0.0°C; control condition). Participants needed to indicate whether they perceived a temperature change or not by pressing the left (“yes”) or right arrow key (“no”). The first half of the experimental trials assessed the unchanged pain responses. In the second half, correct discrimination was either contingently or noncontingently reinforced by monetary rewards. (c) Avoidance task. At the beginning of each trial, participants saw a word pair on the screen in front of them indicating the difficulty of the trial (difficult, easy, or control) and the anticipated intensity of the heat stimulus (high or low). As soon as a blue circle appeared as a target, participants had to react as fast as possible to avoid receiving the announced stimulation. If they reacted while the blue circle was still on the screen, they avoided receiving heat stimulation; if they reacted after the blue circle had already disappeared, they failed and received the heat stimulation. The first half of the experimental trials assessed the unchanged pain responses. In the second half, successful avoidance was either contingently or noncontingently reinforced by monetary rewards.

The within‐subject design included two experimental sessions separated by an interval of approximately 1 week (*M* = 7.98 days, SD = 2.87 days). Each testing session comprised a pre‐assessment of participants' mood and individual pain sensitivity, a main experimental task (in one of the sessions the discrimination task, in the other session the avoidance task), and a post‐assessment of participants' mood and individual pain sensitivity (see Figure 1a). Furthermore, the participants were asked to complete several questionnaires after the first session (see section “Questionnaires”). The order of the two main experimental tasks was counterbalanced across the participants. In both tasks, monetary reinforcement for operant conditioning was implemented after half of the experimental trials. Participants always received contingent reinforcement in one of the tasks and noncontingent reinforcement in the other task. Assignments of these reinforcement conditions were counterbalanced across participants. To avoid confounding reinforcement effects with task order, the study design was fully balanced. That is, approximately a quarter of the participants (*N* = 16) started with the avoidance task and received contingent reinforcement, one quarter (*N* = 15) started with the avoidance task as well but received noncontingent reinforcement, one quarter (*N* = 16) started with the discrimination task and received contingent reinforcement, and one quarter (*N* = 15) started with the discrimination task as well but received noncontingent reinforcement.

### 2.5 Pain Assessments

Before performing the main experimental task, participants' individual heat pain threshold, heat pain tolerance, and perceived intensity and un‐/pleasantness of a series of heat stimuli were assessed as a baseline assessment of the individual pain sensitivity. The pain threshold and tolerance were further used to determine the stimulus intensity of the main experimental tasks to achieve comparable percepts of the painful stimulation across participants.

After the completion of the main experimental task, participants' heat pain threshold, heat pain tolerance, and perceived intensity and un‐/pleasantness of heat stimuli were assessed again to evaluate whether any conditioned effects generalized to self‐reported pain.

#### 2.5.1 Pain Threshold and Tolerance

Each participant's thermal pain threshold and thermal pain tolerance were assessed by the methods of limits (Becker, Ceko, et al. 2013). The temperature of the thermode increased from the baseline temperature (35°C) at a rate of 1°C/s. In the first three runs, the participants indicated their pain threshold by pressing the space bar of the keyboard in front of them. The pain threshold was defined as the point at which the quality of sensation changes and it just starts to hurt. In the subsequent three runs, the participants indicated their pain tolerance by pressing the space bar. The pain tolerance was defined as the point after which the participants could not bear any further increase in temperature. The temperature of the thermode immediately returned to baseline as soon as the space bar was pressed.

#### 2.5.2 Perceived Pain Intensity and Pleasantness

Four different heat stimuli of 3 s duration were each applied twice in a predetermined pseudorandomized order (44°C, 42°C, 48°C, 46°C, 46°C, 48°C, 44°C, 42°C). After each stimulus, participants rated perceived intensity and un‐/pleasantness of the stimulus on the VASs (see “Rating Scales”).

#### 2.5.3 Adjustment of Pain Intensity

To determine the target temperature of the two main experimental tasks, another series of heat stimuli was applied. The length of the heat application was adjusted to match the stimulus during the subsequent main experimental task (discrimination task: 10 s; avoidance task: 3 s). Each heat stimulus was rated by the participants on the intensity VAS. Depending on participants' ratings, the temperature of the following stimulus was adjusted following a staircase procedure upwards or downwards by steps of 0.5°C, aiming at a perceived moderately painful intensity of 130 ± 10 on the VAS for intensity. As soon as participants rated one of the heat stimulations between 120 and 140 on the intensity VAS, the application of further stimuli was stopped (Becker et al. 2015). The resulting temperature was then used as the target stimulus in the discrimination task. For the avoidance task, this temperature served as the individual reference level from which two distinct target temperatures were calculated by adding or subtracting 2°C to define high and low intensity stimuli, respectively.

### 2.6 Experimental Tasks

#### 2.6.1 Discrimination Task

The discrimination task assesses the participants' ability to discriminate small changes in nociceptive input as a surrogate of the sensory‐discriminative pain components.

Each trial began with thermal stimulation increasing from the baseline temperature at a rate of 20°C/s until reaching the individual target temperature (determined in the preassessment of pain sensitivity; see “Pain Assessments”). When the target temperature was reached, it stayed constant for 4 s, after which an exclamation mark appeared as a cue on the screen and remained visible for 500 ms. When this cue disappeared, a variable time interval of 500–1500 milliseconds started after which the temperature either increased by 0.4°C (easy condition), by 0.2°C (difficult condition), or remained unchanged (+0.0°C, control condition). The temperature remained at a plateau after this change for 1 s to decrease afterward back to the target temperature. When the target temperature was reached again and after a fixed interval of 500 ms, participants had 2 s to indicate whether they perceived a temperature change (“yes” or “no”; forced choice) by pressing with the dominant hand the left (“yes”) or the right arrow key (“no”) on the keyboard in front of them. After participants' response, the thermal stimulus returned to baseline at a rate of 20°C/s. The entire thermal stimulation, from ramp‐up to ramp‐down, lasted at maximum 10 s (see Figure 1b). If no response was given, the respective trial was repeated. Participants were not required to specify the direction of the change they perceived.

Every thirteenth trial, participants received a constant stimulation at the target temperature for ten seconds. After the return of this stimulation to the baseline temperature, participants evaluated the perceived pain intensity on the intensity VAS and the perceived un‐/pleasantness on the unpleasantness VAS presented to them on the screen.

Each of the three conditions (easy, difficult, and control) was repeated 40 times, resulting in 120 trials in total. The presentation of these conditions followed intermixed with a predefined pseudo‐randomized order.

Performance of the entire discrimination task lasted approximately 45 min, with short breaks every 15 min, resulting in two breaks. The main outcome measures were the number of correct discriminations and the mean reaction times. The VAS ratings of the intensity and pleasantness of the stimuli were recorded 10 times.

#### 2.6.2 Avoidance Task

The avoidance task is a modified version of the incentive delay task (Gandhi et al. 2013; Knutson et al. 2001), assessing participants' motivation to avoid painful heat stimuli as a surrogate of the emotional–motivational pain components.

Basically, participants' task was to react fast enough to a visual target stimulus to avoid heat stimulation. The difficulty of this avoidance was manipulated with three levels (difficult, easy, and a control condition). In addition, the intensity of the heat stimulus was varied, with high and low intensities. At the start of each trial, a word pair appeared on the screen, indicating both the difficulty of avoiding the heat stimulus (‘easy’, ‘difficult’, or “control”) and the anticipated intensity of this stimulus (‘high’ or “low”). The six possible combinations (easy‐high, easy‐low, difficult‐high, difficult‐low, control‐high, control‐low) were presented equally often in a predefined pseudorandomized order. The word pair was presented for a variable duration between 2000 and 2500 ms, after which a blue circle appeared to signal the target. This variability was introduced to prevent participants from anticipating stimulus onset. Participants were instructed to press the space bar as quickly as possible.

If participants pressed the space bar after the blue circle had disappeared, the heat stimulus was delivered with the intensity indicated before (see Figure 1c). The blue circle disappeared upon keypress or at the end of its presentation time.

The display duration of the blue circle, and with this the difficulty of the avoidance task, was adjusted individually based on reaction times collected during 12 training trials performed at the beginning of the task (Gandhi et al. 2013). In the “easy” condition, the presentation time of the blue circle was 66% of the participant's average baseline reaction time. In the “difficult” condition, the presentation time of the blue circle was 33% of the participant's average baseline reaction time. In the “control” condition, the presentation time of the blue circle was 4 s (no adaptation to individual reaction times). The control condition therefore served as a safe condition with ample time to respond. If participants responded before the target appeared on the screen or failed to respond while the blue circle was displayed, the corresponding trial was repeated.

If participants were not successful in avoiding the heat stimulus, the temperature ramped up from baseline at a rate of 20°C/s to the individually predefined target temperature. As soon as this target temperature was reached, it was held constant for 3 s after which it decreased back to baseline at the same rate. The “high” and “low” stimulation intensities were calculated individually based on the assessed temperature from the adjustment task (see section “Pain Assessments”). Due to a technical error, the two investigators, who performed the testing, systematically applied different temperatures here. One investigator set the “high” intensity corresponding to the temperature determined in the adjustment task and the “low” intensity 2°C below this target temperature (18 participants). The second investigator, set the “low” intensity corresponding to the temperature determined in the adjustment task and the “high” intensity 2°C above this target temperature (44 participants). These target temperatures were based on participants' individually calibrated pain intensity ratings, aiming for a perceived moderate pain level of approximately 130 on a 0–200 VAS scale. We verified that the temperatures used in both conditions remained within the individual pain threshold and tolerance limits assessed before the performance of the avoidance task.

Every 23rd trial, participants received both the “high” and “low” intensity heat stimuli in random order, for 3 s each with no reaction time task related. In these trials, participants had to rate perceived pain intensity and un‐/pleasantness of each stimulus on the respective VAS. Ten such rating trials for each stimulus intensity were performed.

In total, the avoidance task comprised 220 trials. Of these trials, 88 each were of the difficulty “easy” and “difficult”, and 44 were of the condition “control”. Half of the trials (i.e., 110) were of stimulus intensity condition “high” and the other half of “low”. This resulted in each stimulus intensity in 44 difficult and easy trials and 22 control trials. The number of successful avoidances and the mean reaction times were assessed as the main outcome variables. The duration of the task was approximately 45 min. Short breaks of at least 30 s were made every 15 min, resulting in two breaks.

#### 2.6.3 Operant Conditioning by Monetary Rewards

The ability to discriminate small changes in nociceptive input (discrimination task) and the motivation to avoid expected painful stimuli (avoidance task) were both first assessed in their baseline state. After half of the total number of trials for each task (i.e., after 60 trials in the discrimination task and after 110 trials in the avoidance task), these pain responses were either contingently or noncontingently reinforced by monetary rewards (Becker, Gandhi, et al. 2013; Becker, Gandhi, Pomares, et al. 2017; Gandhi et al. 2013). When contingently reinforced, each correct discrimination and each successful avoidance was continuously rewarded with CHF 0.20, which was displayed on the screen in front of the participant immediately after the respective pain response was given (Becker, Gandhi, Chen, and Schweinhardt 2017). Noncontingent rewards were used as a control condition and implemented by a yoked control procedure: the participants received the rewards in the order the penultimate participant had received the rewards in their contingent reinforcement condition. Since the mere change of winning, even with random rewards, is known to increase the motivation to work for such rewards (Clark et al. 2009; Dong et al. 2014), these noncontingent rewards were aimed to achieve a comparable motivational drive as with the contingent condition.

The total win that the participants achieved during the experimental tasks was paid to them in cash at the end of their last testing session. Participants received a fixed reimbursement of 20 CHF for their participation in the study. In addition, participants were paid the summed‐up total win they achieved during both experimental tasks in cash at the end of their last testing session. Across all participants, these net wins ranged from 15.40 to 31.20 CHF based on individual performance.

### 2.7 Questionnaires

Participants were asked to fill in several questionnaires using German or English versions depending on participants' language preferences. The selection of questionnaires was based on their relevance to individual differences in affective, cognitive, and motivational processes that could influence pain perception and reinforcement learning. While no specific hypotheses were formulated for each questionnaire, they were included for exploratory purposes to assess potential relationships between these psychological factors and task performance.

#### 2.7.1 Positive and Negative Affect Schedule (PANAS)

The PANAS is a self‐report questionnaire consisting of 12 adjectives of different sensations and feelings. For each adjective, the participants are asked to indicate on a five‐point Likert scale from 1 = “not at all” to 5 = “very much” to which extent they are feeling that way at the given time. Ten adjectives measure the dimension of positive affect; 10 adjectives measure the dimension of negative affect (Watson et al. 1988; Krohne et al. 1996). Using the PANAS allowed monitoring whether participants' mood changed during a session or differed between the sessions and whether participants' mood was related to the pain responses (Goli et al. 2016; Karsdorp et al. 2013; Ossipov et al. 2010).

#### 2.7.2 Fear of Pain Questionnaire‐III (FPQ‐III)

The FPQ‐III measures pain‐related fear with thirty items, each representing a situation usually causing pain (e.g., “Being in an automobile accident.”). Participants are asked to rate their fear of pain for each situation on a five‐point Likert scale from 1 = “not afraid at all” to 5 = “extremely afraid”. The items are further divided into three subscales with ten items each for severe pain, minor pain, and medical pain (McNeil and Rainwater 1998). As no validated German version of the FPQ‐III was available, we used a translated version that was created using a forward‐backward translation procedure to ensure conceptual and linguistic equivalence.

#### 2.7.3 Fear Avoidance Beliefs Questionnaire (FABQ)

The FABQ measures fear of pain and resulting avoidance of physical activity or work. In particular, the FABQ focuses on how fear avoidance belief affects or would affect low back pain. Therefore, the participants rate 16 items on a seven‐point Likert scale from 0 = “completely disagree” to 6 = “completely agree”. The first five items relate to physical activity (e.g., “My pain was caused by physical activity”), while the remaining eleven items refer to work (e.g., “My pain was caused by my work or by an accident at work.”); they are further divided into two subscales: the fear‐avoidance beliefs about physical activity (four items), and the fear‐avoidance beliefs about work (seven items) (Waddell et al. 1993; Pfingsten et al. 2000).

#### 2.7.4 Pain Catastrophizing Scale (PCS)

The PCS, consisting of 13 items describing thoughts or feelings when experiencing pain, provides a valid index of catastrophizing. Using a five‐point Likert scale from 0 = “not at all” to 4 = “all the time”, participants are asked to indicate the degree to which they have the described thoughts and feelings when they are experiencing pain (e.g., “I worry all the time about whether the pain will end.”). There are three subscales assessing rumination (four items), magnification (three items), and helplessness (six items) (Sullivan et al. 1995; Meyer et al. 2008).

#### 2.7.5 Need Inventory of Sensation Seeking (NISS)

The NISS conceptualises sensation seeking as a motivational trait, that is, the persistent, dispositional need for stimulation. Participants are asked to rate 17 items on how often they have felt this way in the past 6 months on a five‐point Likert scale from 1 = “almost never” to 5 = “almost always” (e.g., “I like to find myself in situations which make my heart beat faster.”) (Roth and Hammelstein 2012).

#### 2.7.6 Beck Depression Inventory‐II (BDI‐II)

The BDI‐II is widely used to measure the presence and severity of depressive symptoms. Participants are asked to respond to each of the 21 questions on a four‐point scale about the occurrence of the described depressive symptomatology in the previous 2 weeks (e.g., “I do not feel sad./I feel sad./I am sad all the time and I cannot snap out of it./I am so sad and unhappy that I can't stand it.”) (Beck et al. 1996; Kühner et al. 2007).

#### 2.7.7 State–Trait Anxiety Inventory (STAI)

The STAI can be used to assess trait and state anxiety. It consists of 20 items referring to trait anxiety (e.g., “I feel calm.”) and 20 items referring to state anxiety (e.g., “I feel pleasant.”). All 40 statements are rated on a four‐point Likert scale from 1 = “almost never” to 4 = “almost always” (Spielberger et al. 1983; Grimm 2009).

#### 2.7.8 Revised Life Orientation Test (LOT‐R)

The LOT‐R assesses dispositional optimism. Participants are asked to answer 10 statements (e.g., “In uncertain times, I usually expect the best.”) by indicating the extent of agreement using a five‐point Likert scale from 0 = “strongly disagree” to 4 = “strongly agree” (Scheier et al. 1994; Herzberg et al. 2006).

#### 2.7.9 Snaith‐Hamilton Pleasure Scale (SHAPS)

The SHAPS is used to assess the individual hedonic capacity, i.e., the ability to experience pleasure (e.g., “I would enjoy my favorite television or radio program.”). It consists of 14 items with a set of four response categories “definitely agree”, “agree”, “disagree”, and “strongly disagree”, with either of the agree responses receiving a score of 0 and with either of the disagree responses receiving a score of 1 (Snaith et al. 1995; Franz et al. 1998).

#### 2.7.10 Fifteen‐Item Short Form of the Barratt Impulsiveness Scale (BIS‐15)

The BIS‐15 measures the personality trait of impulsivity with five items for each of the three subscales non‐planning (e.g., “I plan for job security.”), motor (e.g., “I act on impulse.”), and attentional impulsivity (e.g., “I am restless at lectures or talks.”). Each item is rated on a four‐point Likert scale from 1 = “rarely/never” to 4 = “almost always/always” (Spinella 2007; Meule et al. 2001).

### 2.8 Data Analysis

Outliers in the data were identified using the exclusive interquartile range method and removed from the statistical analyses. In the avoidance task, in the reaction times 1110 out of 13,424 data points were identified as outliers and removed; there were no outliers in success rates (out of 732 data points). In the discrimination task, in the reaction times 249 out of 7316 data points and for the success rates 17 out of 368 data points were identified as outliers and removed. For both, the discrimination and avoidance tasks, reaction times from both correct and incorrect trials were included in the analysis. This approach was chosen because we were interested in how reaction times changed over time, regardless of the correctness of the responses. Outliers were excluded from the analysis to ensure data validity.

Reaction times were analysed using linear mixed model designs (lmerTest R package). This approach was chosen because it allows for the estimation of fixed effects while accounting for random effects, such as individual differences in baseline reaction times. This flexibility is crucial for accurately modelling our complex, repeated measures data, where individual participants contribute multiple data points across different conditions. Post hoc pairwise comparisons were conducted with Tukey adjusted *p*‐values. To address concerns regarding the distribution of reaction times and the assumptions of normality, we conducted diagnostic checks. First, we examined the raw reaction times distribution for both tasks. The avoidance task showed a relatively mild right skew (skewness = 0.93), whereas the discrimination task exhibited a more pronounced right‐skewed distribution (skewness = 1.03). These findings are consistent with previous reports that reaction times tend to follow right‐skewed distributions due to their positive‐bound nature and variability across trials (e.g., Rousselet and Wilcox 2020). We also assessed the normality of residuals using Q–Q plots, which revealed some deviations from normality, particularly at the tails, for both tasks. However, the central portions of the residuals showed good alignment with the expected normal distribution. Given these results, we chose to retain our original linear mixed model approach, as the deviations from normality were not substantial enough to necessitate the use of a generalised linear mixed model. The linear mixed model is robust to moderate non‐normality and provides interpretable estimates of mean reaction time differences, accounting for random intercepts.

The numbers of successful answers were analysed using generalised linear mixed model designs (lme4 R package) as the depended variable of success is binarily coded (0 = “failure”, 1 = “success”), followed up with post‐hoc pairwise comparisons with Tukey adjusted *p*‐values. To characterize the effects of the main factors of interest, these main factors were first analyzed in the simplest model possible with the main factors only, that is, the within‐subject factor “phase” (baseline vs. learning) and the between‐subject factor “contingency” (contingent vs. noncontingent). Afterwards, additional factors were included to test whether they have significant effects on the main interventions. By including all other factors in all combinations and comparing the models with ANOVA, the model with the best parsimonious fit was selected. These additional factors were for the discrimination task the within‐subject factor “difficulty” (easy vs. difficult vs. control) and for the avoidance task the within‐subject factors “difficulty” (difficult vs. easy vs. control) and “intensity” (low vs. high) and the between‐subject factor “temperature” (+2°C vs. −2°C).

For the VAS intensity ratings 4 out of 1222 data points and for the unpleasantness ratings 2 out of 1222 data points were removed as outliers in the avoidance task and in the discrimination task 14 out of 608 data points for the intensity ratings and 51 out of 608 data points for the unpleasantness ratings. All VAS intensity and pleasantness ratings were analysed using a linear mixed model design (lmerTest R package). Since the VAS ratings assess subjective pain perception, which can be influenced by various experimental conditions, it was essential to include and test multiple factors in our analysis. In the avoidance task, additionally to the main factors of interest “phase” (within‐subject; baseline vs. learning) and “contingency” (between‐subject; contingent vs. noncontingent), all other factors in all combinations were included and the models were compared with ANOVA to select the best parsimonious fit. These additional factors were the within‐subject factor “intensity” (low vs. high) and the between‐subject factor “temperature” (+2°C vs. −2°C). Post hoc pairwise comparisons were conducted with Tukey adjusted *p*‐values.

Pain threshold and pain tolerance were analysed separately of the experimental task calculating the mean of three runs each in the pre‐ and post‐measurement. In the avoidance task, there were no outliers in pain thresholds out of 120 data points; for the pain tolerance 5 out of 120 data points were removed as outliers. In the discrimination task, for the pain thresholds 1 of 120 data points was removed as outliers; for the pain tolerance 10 out of 120 data points were removed as outliers. The pain threshold and pain tolerance were analysed using a linear mixed model design (lmerTest R package) with the main factors of interest “time” (within‐subject; pre‐ vs. post‐measurement) and “contingency” (between‐subject; contingent vs. noncontingent). Post hoc pairwise comparisons were conducted with Tukey adjusted *p*‐values.

The scores of all questionnaires were analysed exploratory for correlations with the changes in reaction times and success rates from the baseline to the learning phase in contingently reinforced discrimination and avoidance tasks. For these correlations, all *p*‐values were adjusted for multiple testing using the Bonferroni correction. In a subsequent analysis, independent *t*‐tests were conducted to examine whether the contingency groups (contingent reinforcement in the avoidance task vs. contingent reinforcement in the discrimination task) differed in the traits that showed significant correlations in the previous analysis.

The significance level was set to 5%. Effect sizes were reported by means of partial eta‐squared $\eta_{p}^{2}$ (small: $\eta_{p}^{2}$ > 0.01, medium: $\eta_{p}^{2}$ > 0.06, large: $\eta_{p}^{2}$ > 0.14), Odds‐Ratio (OR), 95% bootstrap confidence intervals (CI; nsim = 100) and Cohen's *d* (small: *d* = 0.2, medium: *d* = 0.5, large: *d* = 0.8). All statistical analyses were performed using RStudio (R Core Team 2022).

## 3 Results

### 3.1 Contingent Operant Reinforcement Decreased Reaction Times and Increased Success Rates of Pain Avoidance Behaviour

**FIGURE 2:**
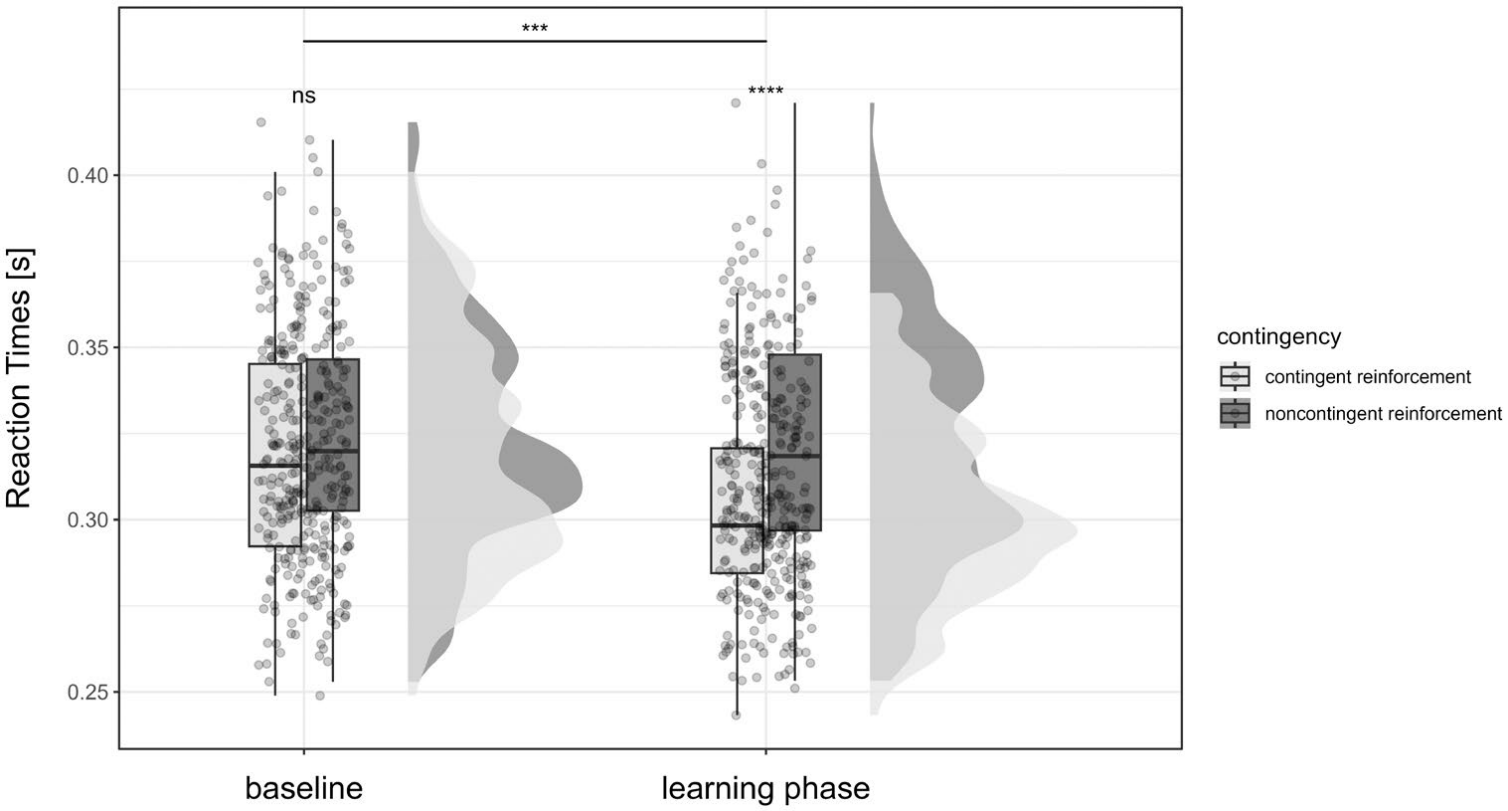
Reaction times during the avoidance task. Reaction times in seconds in the avoidance task in the baseline and learning phase for contingent and noncontingent reinforcement in the learning phase. Dots represent the mean reaction times per participant, the box bounds the IQR divided by the median, and Tukey‐style whiskers extending to a maximum of 1.5 × IQR. Half violins indicate the distributions of the mean reaction times. Left: Contingent reinforcement; Right: Noncontingent reinforcement. ****p < 0.0001, ***p < 0.001, ns: Not significant.

**FIGURE 3:**
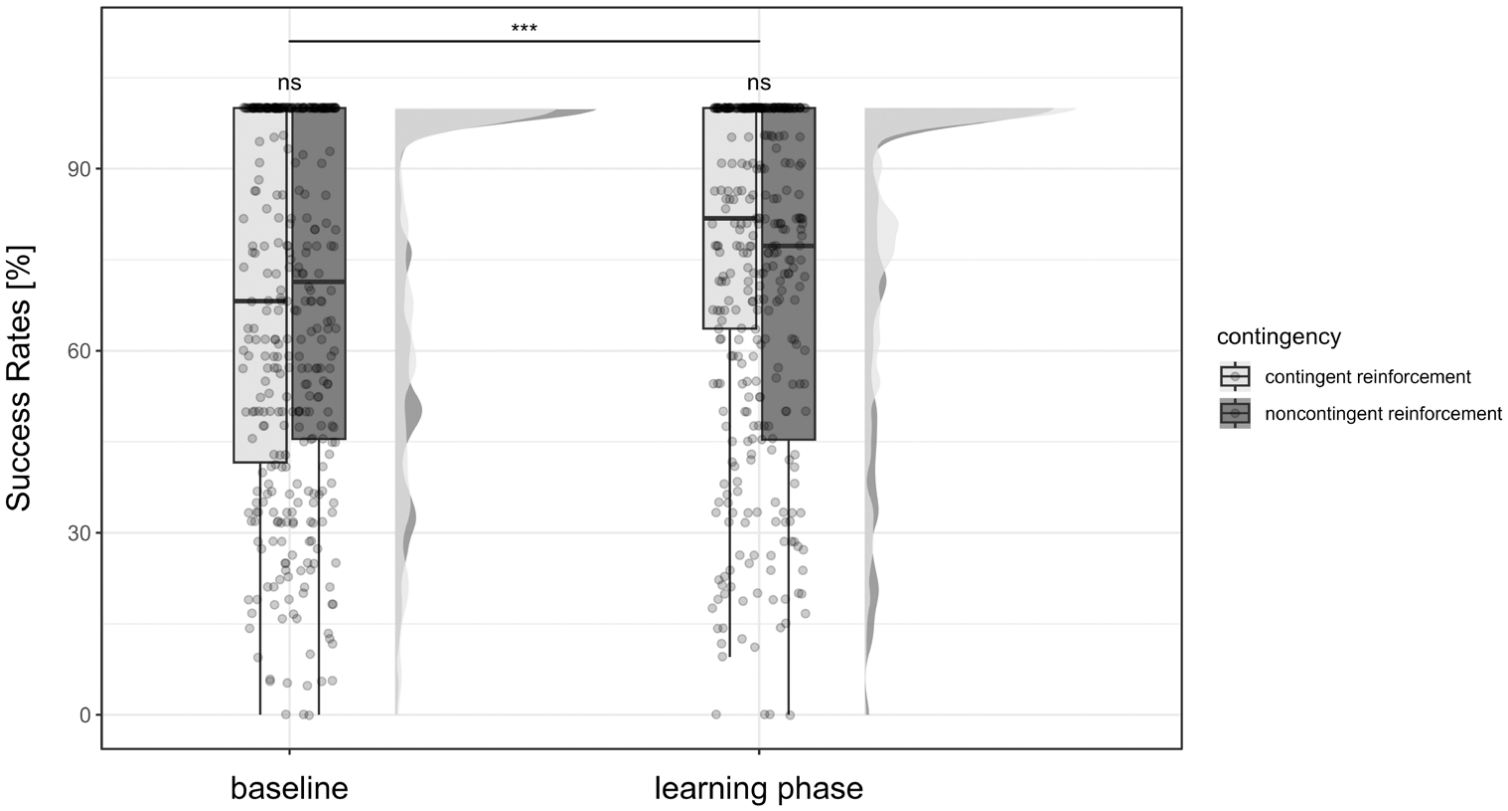
Success rates during the avoidance task. Success rates in percent in the avoidance task in the baseline and learning phase for contingent and noncontingent reinforcement. Dots represent the mean reaction times per participant, the box bounds the IQR divided by the median, and Tukey‐style whiskers extending to a maximum of 1.5 × IQR. Half violins indicate the distributions of the mean reaction times. Left: contingent reinforcement; Right: noncontingent reinforcement. ***p < 0.001, ns: not significant.

As hypothesized, contingent operant reinforcement significantly decreased reaction times (Figure 2; *F*_(1)_ = 8.89, *p* = 0.004, $\eta_{p}^{2}$ = 0.13) and increased success rates (Figure 3; $\chi_{\left(1\right)}^{2}$ = 32.02, *p* < 0.001, OR = 1.13 [95% CI: 0.40 to 0.86]) in the learning phase compared to baseline in the avoidance task. This simple model, which tested the main effects of phase and contingency only, is detailed in Appendix S1.

After testing and confirming the main effects of phase and contingency on reaction times, further factors were included based on the best fitting model, which was the one with the additional within‐subject factors “difficulty” (difficult vs. easy vs. control) and “intensity” (low vs. high). Any model including the between‐subject factor “temperature” (+2°C vs. −2°C) did not fit better in explaining the variance in reaction times. Effects of phase and contingency of the simple model were confirmed (see Table 1), with additional significant main effects of “difficulty” (*F*_(2)_ = 97.73, *p* < 0.001, $\eta_{p}^{2}$ = 0.02) and “intensity” (*F*_(1)_ = 14.22, *p* < 0.001, $\eta_{p}^{2}$ < 0.01). Regarding the effects of difficulty, post hoc comparisons showed that reaction times were faster in the difficult compared to the easy (*p* < 0.001, *d* = −0.21) and compared to the control condition (*p* < 0.001, *d* = −0.31), as expected. Reactions were also significantly shorter in the easy compared to the control condition (*p* = 0.0002, *d* = −0.10). Like difficulty, intensity of the stimulation affected reaction times with faster reactions with high compared to low stimulation intensities (*p* = 0.0002, *d* = −0.07). These results indicate that the reaction times were faster with increasing difficulty and high stimulation intensities, although difficulty and intensity did not interaction and had no additional effect on operant conditioning.

**TABLE 1:** Complex model of the reaction times in the avoidance task.

	Sum Sq	Mean Sq	NumDF	DenDF	*F* value	Pr (<*F*)
Phase	0.02662	0.026621	1	60.3	13.6943	0.0004675***
Contingency	0.00511	0.005111	1	59.1	2.6291	0.1102502
Difficulty	0.37997	0.189983	2	12173.2	97.7294	< 2.2 e‐16***
Intensity	0.02764	0.027643	1	12173.1	14.2199	0.0001634***
Phase × contingency	0.01727	0.017272	1	60.3	8.8850	0.0041412**
Phase × difficulty	0.00029	0.000144	2	12173.9	0.0740	0.9286561
Contingency × difficulty	0.00096	0.000481	2	12173.2	0.2476	0.7806473
Phase × intensity	0.00074	0.000745	1	12173.1	0.3832	0.5359316
Contingency × intensity	0.00139	0.001393	1	12173.1	0.7166	0.3972659
Difficulty × intensity	0.00412	0.002062	2	12173.0	1.0606	0.3462780
Phase × contingency × difficulty	0.00002	0.000012	2	12173.9	0.0064	0.9936367
Phase × contingency × intensity	0.00061	0.000608	1	12173.1	0.3125	0.5761542
Phase × difficulty × intensity	0.00727	0.003633	2	12172.7	1.8689	0.1543417
Contingency × difficulty × intensity	0.00329	0.001644	2	12173.0	0.8455	0.4293845
Phase × contingency × difficulty × intensity	0.00870	0.004351	2	12172.7	2.2384	0.1066752

*Note:* Complex model of the reaction times in the avoidance task with the factors phase (baseline vs. learning phase), contingency (contingent vs. noncontingent), difficulty (difficult vs. easy vs. control) and intensity (low vs. high).

***p < 0.001, **p < 0.01.

For the number of successful reactions, the best fitting model with additional factors was the one with the within‐subject factors “difficulty” (difficult vs. easy vs. control) and “intensity” (low vs. high). Any model including the between‐subject factor “temperature” (+2°C vs. −2°C) did not fit better. This complex model confirmed the interaction effect of the simple model between phase and contingency (see Table 2), with additional significant main effects of “difficulty” ($\chi_{\left(2\right)}^{2}$ = 33.1067, *p* < 0.001, OR easy = 2.03 [95% CI: 1.59 to 2.59]). The number of successful reactions were significantly lower in the difficult compared to the easy trials (*p* < 0.001, *d* = −0.18), as expected, although there was no significant difference between the difficult and control condition (*p* > 0.999, *d* = −0.52). Similarly, number of successful avoidances shows no significant difference between the easy and control conditions (*p* > 0.999, *d* = −0.34).

**TABLE 2:** Complex model of the success rates in the avoidance task.

	Chisq	Df	Pr (>Chisq)
Intercept	2.1037	1	0.1469410
Phase	13.7036	1	0.0002140***
Contingency	0.8402	1	0.3593520
Difficulty	33.1067	2	6.471 e‐08***
Intensity	2.1511	1	0.1424640
Phase × contingency	7.5430	1	0.0060250**
Phase × difficulty	1.6854	2	0.4305400
Contingency × difficulty	0.7608	2	0.6835740
Reinforcement × intensity	1.3447	1	0.2462020
Contingency × intensity	1.2638	1	0.2609290
Difficulty × intensity	0.0234	2	0.988381
Phase × contingency × difficulty	0.2486	2	0.8830980
Phase × contingency × intensity	0.2223	1	0.637273
Phase × difficulty × intensity	1.9161	2	0.383642
Contingency × difficulty × intensity	2.2277	2	0.328287
Phase × contingency × difficulty × intensity	0.5164	2	0.772445

*Note:* Complex model of the success rates in the avoidance task with the factors phase (baseline vs. learning phase), contingency (contingent vs. noncontingent), difficulty (difficult vs. easy. vs. control) and intensity (low vs. high).

***p < 0.001, **p < 0.01.

#### 3.1.1 Contingent Operant Reinforcement Did Not Affect Reaction Times and Success Rates of Discriminative Pain Behaviour Differentially Compared to Noncontingent Reinforcement

**FIGURE 4:**
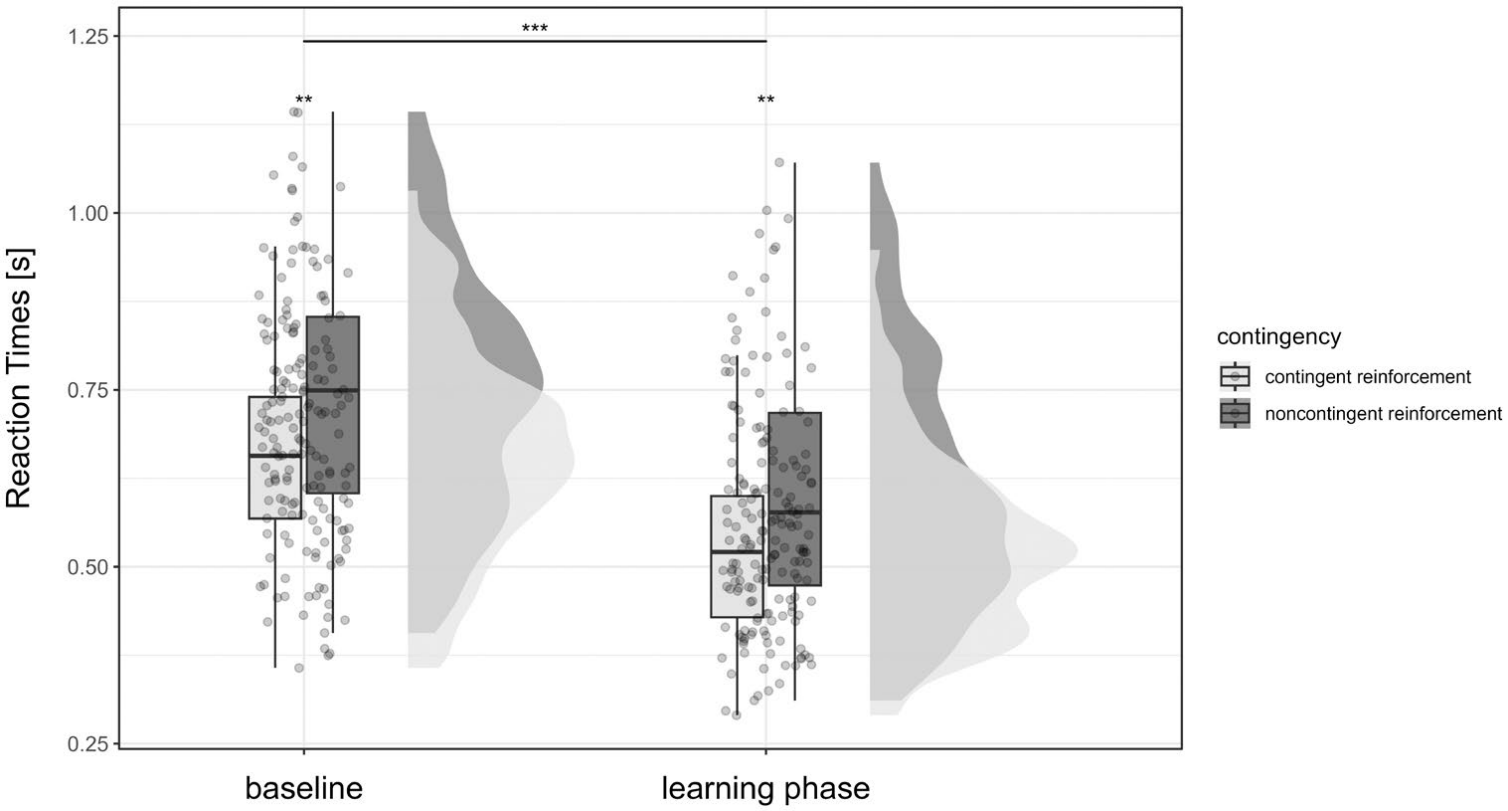
Reaction times during the discrimination task. Reaction times in seconds in the discrimination task in the baseline and learning phases for contingent and noncontingent reinforcement in the learning phase. Dots represent the mean reaction times per participant, the box bounds the IQR divided by the median, and Tukey‐style whiskers extending to a maximum of 1.5 × IQR. Half violins indicate the distributions of the mean reaction times. Left: contingent reinforcement; Right: noncontingent reinforcement. ***p < 0.001, **p < 0.01.

**FIGURE 5:**
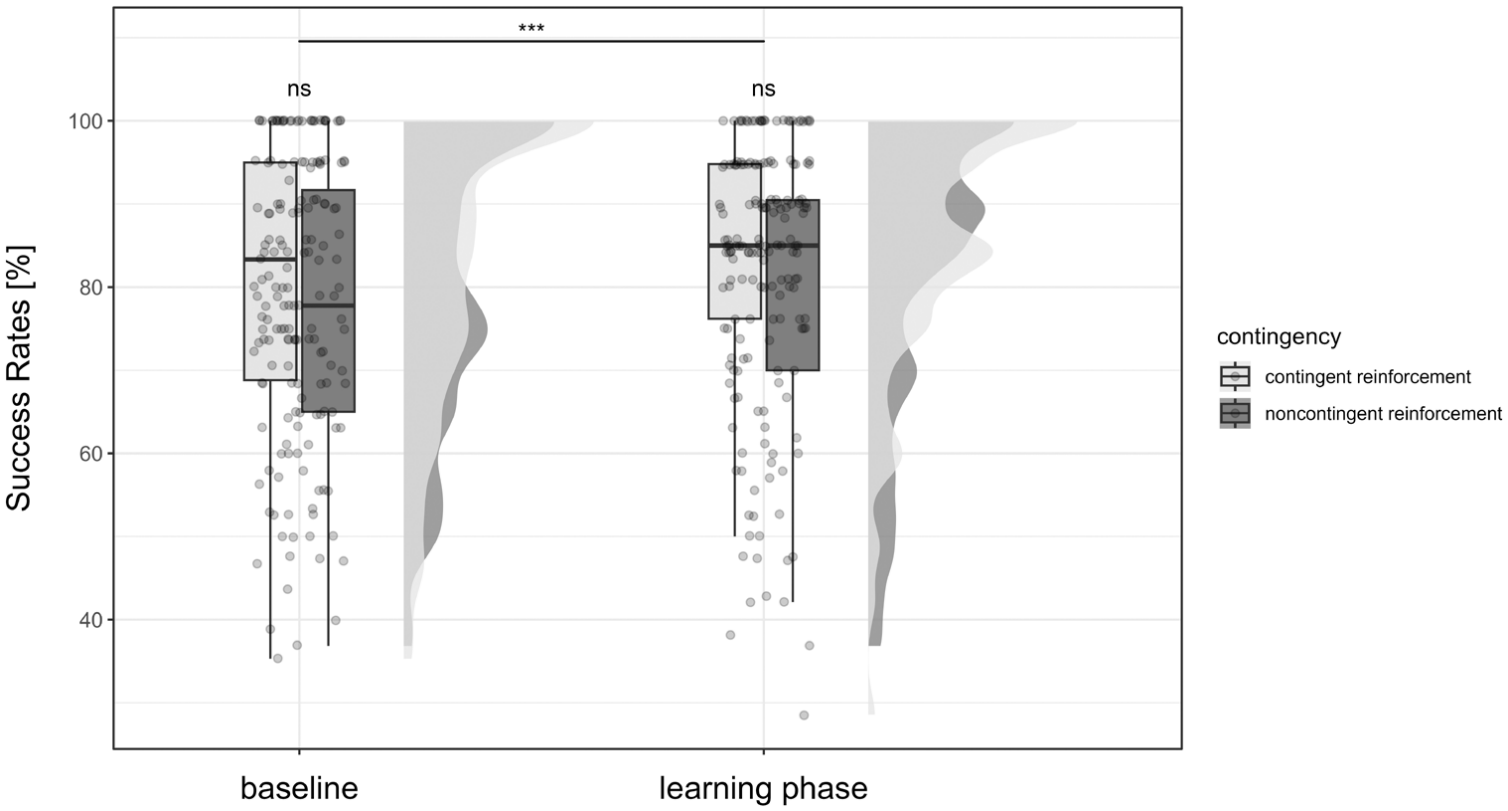
Success rates during the discrimination task. The success rates in percent in the discrimination task in the baseline and learning phase for contingent and noncontingent reinforcement. The dots represent the mean reaction times per participant, the box bounds the IQR divided by the median, and Tukey‐style whiskers extend to a maximum of 1.5 × IQR beyond the box. Half violins show the distribution of the mean reaction times. ***p < 0.001, ns, Not significant.

In contrast to the avoidance task, reaction times in the discrimination task increased from baseline to the learning phase, with no significant interaction between phase and contingency (Figure 4; *F*_(1)_ = 0.13, *p* = 0.718, $\eta_{p}^{2}$ < 0.01). Similarly, the number of correct answers was higher in the learning phase compared to baseline with no differences for contingent and noncontingent reinforcement (Figure 5; $\chi_{\left(1\right)}^{2}$ = 0.079, *p* = 0.778, OR = 1.23 [95% CI: 0.81 to 1.36]). Complete results from the simple model, which tested the main effects of phase and contingency only, are detailed in Appendix S1.

To test for more complex effects on reaction times, the best fitting model was the one with the additional within‐subject factor “difficulty” (control vs. easy vs. difficult). This more complex model confirmed the effects of “phase” and “contingency” of the simple model (see Table 3). In addition, task difficulty affected reaction times in that reactions were faster in the easy compared to difficult condition, but only in the learning phase (interaction effect: phase × difficulty: *F*_(2)_ = 5.86, *p* = 0.003, $\eta_{p}^{2}$ < 0.01; post hoc pairwise comparison: easy vs. difficult, learning phase: *p* = 0.002, *d* = 0.16, baseline: *p* = 0.875, *d* = 0.05). In the control condition, reaction times were significantly slower compared to the easy and difficult trials during baseline (easy: *p* < 0.001, *d* = 0.58; difficult: *p* < 0.001, *d* = 0.54) and the learning phase (easy: *p* < 0.001, *d* = 0.49; difficult: *p* < 0.001, *d* = 0.34).

**TABLE 3:** Complex model of the reaction times in the discrimination task.

	Sum Sq	Mean Sq	NumDF	DenDF	*F* value	Pr (<*F*)
Phase	7.8718	7.8718	1	59.2	130.2342	< 2.2 e‐16***
Contingency	0.3462	0.3462	1	62.9	5.7271	0.019703*
Difficulty	23.0559	11.5279	2	6938.4	190.7233	< 2.2 e‐16***
Phase × contingency	0.0096	0.0096	1	59.6	0.1592	0.691337
Phase × difficulty	0.7088	0.3544	2	6938.6	5.8636	0.002855**
Contingency × difficulty	0.0237	0.0118	2	6938.4	0.1960	0.822041
Phase × contingency × difficulty	0.22200	0.111	2	6938.6	1.8366	0.159430

*Note:* Complex model of the reaction times in the discrimination task with the factors phase (baseline vs. learning phase), contingency (contingent vs. noncontingent) and difficulty (control vs. easy vs. difficult).

***p < 0.001, **p < 0.01, *p < 0.05.

For the number of correct answers, the best fitting model was the one with the additional within‐subject factor “difficulty” (control vs. easy vs. difficult). The complex model confirmed the results of the simple model only on “phase” (see Table 4). The difficulty of the task modulated the number of correct answers as indicated by a significant main effect of “difficulty” ($\chi_{\left(2\right)}^{2}$ = 122.93, *p* < 0.001, OR easy = 0.49 [95% CI: 3.70 to 6.28], OR difficult = 0.60 [95% CI: 2.13 to 3.34]). As intended, number of correct answers were higher in the easy compared to the difficult and control conditions (difficult: *p* < 0.001, *d* = −0.07; control: *p* < 0.001, *d* = −0.24). The number of correct answers were higher in the difficult compared to the control conditions (*p* < 0.001, *d* = −0.17). Moreover, task difficulty interacted with phase (interaction effect: “phase” × “difficulty”: $\chi_{\left(2\right)}^{2}$ = 13.60, *p* = 0.001, OR easy = 1.14 [95% CI: 0.36 to 0.68], OR difficult = 1.33 [95% CI: 0.42 to 0.86]). Specifically, correct answers were more frequent in the learning phase compared to baseline but only for the control condition (post hoc comparisons: control: *p* < 0.001, *d* = −0.12; easy: *p* = 0.926, *d* = −0.01; difficult: *p* = 0.929, *d* = −0.02). These results suggest that learning effects occurred only in control trials, but not in the easy and difficult trials.

**TABLE 4:** Complex model of the success rates in the discrimination task.

	Chisq	Df	Pr (>Chisq)
Intercept	6.0131	1	0.0142*
Phase	21.5714	1	3.409 e‐06***
Contingency	0.2783	1	0.5978270
Difficulty	122.9277	2	< 2.2 e‐16
Phase × contingency	0.5912	1	0.441958
Phase × difficulty	13.5981	2	0.001115**
Contingency × difficulty	2.6601	2	0.264463
Phase × contingency × difficulty	3.4368	2	0.1793570

*Note:* Complex model of the success rates in the discrimination task with the factors phase (baseline vs. learning phase), contingency (contingent vs. noncontingent) and difficulty (control vs. easy vs. difficult).

***p < 0.001, **p < 0.01, *p < 0.05.

#### 3.1.2 VAS Ratings of Stimulus Intensity and Unpleasantness Do Not Change Over the Course of the Avoidance and the Discrimination Task

##### 3.1.2.1 Avoidance Task

Table 5 presents the absolute values of intensity and unpleasantness ratings the participants gave during the avoidance task for low and high stimulus intensities during the baseline as well as during the learning phase.

**TABLE 5:** Absolute values of the intensity and unpleasantness ratings in the avoidance task.

		Low intensity		High intensity	
		Contingent	Noncontingent	Contingent	Noncontingent
*Intensity*					
−2°C	Baseline	84.80 (32.43)	76.30 (28.88)	126.32 (25.58)	121.42 (22.16)
	Learning phase	84.09 (34.54)	76.99 (25.41)	123.03 (28.08)	119.17 (21.31)
+2°C	Baseline	75.88 (28.89)	75.98 (23.78)	110.62 (26.59)	98.31 (27.94)
	Learning phase	79.52 (33.41)	73.53 (30.18)	113.76 (33.53)	101.68 (35.97)
*Un‐/pleasantness*					
−2°C	Baseline	6.53 (17.06)	19.68 (12.04)	−6.89 (14.72)	−2.64 (16.32)
	Learning phase	9.05 (17.52)	21.59 (14.41)	−7.54 (15.63)	0.66 (11.02)
+2°C	Baseline	11.10 (19.34)	14.96 (17.77)	−8.14 (15.08)	0.83 (19.23)
	Learning phase	11.84 (22.24)	14.75 (15.67)	−3.10 (21.16)	0.69 (15.00)

*Note:* Mean and standard deviations (in parenthesis) of intensity and un‐/pleasantness ratings for low and high stimulus intensities in the avoidance task in the baseline and the learning phase for the groups with contingent and non‐contingent reinforcement.

Model comparisons showed that the best fitting model for the intensity ratings included the within‐subject factors “phase” (baseline vs. reinforced) and “intensity” (low vs. high), the between‐subject factors “contingency” (contingent vs. noncontingent) and “temperature” (+2°C vs. −2°C), and their interactions. While intensity ratings did not differ significantly between baseline and learning phase (main effect “phase”: *F*_(1)_ = 0.01, *p* = 0.906, $\eta_{p}^{2}$ < 0.01) and between contingent and noncontingent reinforcement (main effect “contingency”: *F*_(1)_ = 0.74, *p* = 0.395, $\eta_{p}^{2}$ = 0.01; interaction effect “phase” × “contingency”: *F*_(1)_ = 0.03, *p* = 0.853, $\eta_{p}^{2}$ < 0.01), they differed for high and low stimulation intensities (main effect “intensity”: *F*_(1)_ = 441.65, *p* < 0.001, $\eta_{p}^{2}$ = 0.29; Table 6), as expected. The factor “temperature,” that is, the two subgroups with +2°C and −2°C in the determination of the stimulation intensities, did not show a main effect but interacted with the stimulation intensity as a factor (*F*_(1)_ = 13.25, *p* < 0.001, $\eta_{p}^{2}$ = 0.03). For both the +2°C and the −2°C subgroups, ratings were lower for low vs. high stimulation intensities (post hoc comparisons: +2°C: *p* < 0.001, *d* = 1.10; −2°C: *p* < 0.001, *d* = 1.55). Post hoc comparisons confirmed no significant difference between the +2°C and −2°C subgroups neither for the low (*p* = 0.951, *d* = 0.15) nor for the high stimulation intensity (*p* = 0.138, *d* = 0.61). The lack of a difference between the +2°C and −2°C subgroups is surprising, because they differed on average by 2°C in the absolute temperatures (+2°C subgroup: low = 46.09°C ± 1.12°C, high = 47.78°C ± 1.01°C; −2°C subgroup: low = 44.26 ± 1.16°C, high = 46.26°C ± 1.16°C), suggesting that the relative difference between the applied intensities might had a stronger influence on intensity ratings than the absolute temperatures.

**TABLE 6:** Complex model of the intensity ratings in the avoidance task.

	Sum Sq	Mean Sq	NumDF	DenDF	*F* value	Pr (<*F*)
Phase	11	11	1	57.19	0.0141	0.9059459
Contingency	549	549	1	57.00	0.7350	0.3948425
Temperature	1455	1455	1	57.00	1.9477	0.1682507
Intensity	330,019	330,019	1	1092.13	441.6527	< 2.2 e‐16***
Phase × contingency	26	26	1	57.19	0.0348	0.8526637
Phase × temperature	562	562	1	57.19	0.7521	0.3894317
Contingency × temperature	9	9	1	57.00	0.0115	0.9150744
Phase × intensity	17	17	1	1092.13	0.0229	0.8796417
Contingency × intensity	354	354	1	1092.13	0.4741	0.4912496
Temperature × intensity	9900	9900	1	1092.13	13.2495	0.0002854***
Phase × contingency × temperature	34	34	1	57.19	0.0451	0.8325512
Phase × contingency × intensity	56	56	1	1092.13	0.0749	0.7843674
Phase × temperature × intensity	696	696	1	1092.13	0.9314	0.3347078
Contingency × temperature × intensity	2975	2975	1	1092.13	3.9808	0.0462707*
Phase × contingency × temperature × intensity	308	308	1	1092.13	0.4124	0.5209027

*Note:* Complex model of the intensity ratings in the avoidance task with the factors phase (baseline vs. learning phase), intensity (low vs. high), contingency (contingent vs. noncontingent), temperature (+2°C vs. −2°C).

***p < 0.001, *p < 0.05.

Similar to the intensity ratings, model comparisons showed that the best fitting model for the unpleasantness ratings included the within‐subject factors “phase” (baseline vs. reinforced) and “intensity” (low vs. high), the between‐subject factors “contingency” (contingent vs. noncontingent) and “temperature” (+2°C vs. −2°C), and their interactions. In contrast to the intensity ratings, unpleasantness ratings showed a difference between contingent and non‐contingent reinforcement (main effect “contingency”: *F*_(1)_ = 5.17, *p* = 0.027, $\eta_{p}^{2}$ = 0.08; Table 7), although with no difference between baseline and learning phase (main effect “phase”: *F*_(1)_ = 0.596, *p* = 0.443, $\eta_{p}^{2}$ = 0.01; interaction “phase” × “contingency”: *F*_(1)_ = 0.15, *p* = 0.705, $\eta_{p}^{2}$ < 0.01). As for the intensity ratings, unpleasantness ratings were significantly different for high and low stimulation intensities (main effect of “intensity”: *F*_(1)_ = 205.82, *p* < 0.001, $\eta_{p}^{2}$ = 0.16), which interacted with the factor “temperature”, that is, the +2°C and –2°C subgroups (interaction “temperature” × “intensity”: *F*_(1)_ = 12.93, *p* < 0.001, $\eta_{p}^{2}$ = 0.01). Post hoc comparisons indicated that the stimuli were perceived as more unpleasant with high compared to low stimulation intensities in both the +2°C (*p* < 0.001, *d* = −0.68) and the −2°C subgroup (*p* < 0.001, *d* = −1.13). As for the intensity ratings, unpleasantness ratings did not differ significantly between the +2°C and −2°C subgroup for the low (*p* = 0.426, *d* = 0.38) and high stimulation intensities (*p* = 0.993, *d* = −0.07).

**TABLE 7:** Complex model of the un‐/pleasantness ratings in the avoidance task.

	Sum Sq	Mean Sq	NumDF	DenDF	*F* value	Pr (<*F*)
Phase	432	432	1	57.05	0.5964	0.4431553
Contingency	3741	3741	1	57.00	5.1673	0.0268033*
Temperature	304	304	1	57.00	0.4196	0.5197476
Intensity	149,024	149,024	1	1091.92	205.8195	< 2.2 e‐16***
Phase × contingency	105	105	1	57.05	0.1452	0.7046244
Phase × temperature	468	468	1	57.05	0.6461	0.4248597
contingency × temperature	430	430	1	57.00	0.5940	0.4440681
Phase × intensity	63	63	1	1091.92	0.0874	0.7675282
Contingency × intensity	1188	1188	1	1091.92	1.6408	0.2004928
Temperature × intensity	9360	9360	1	1091.92	12.9268	0.0003384***
Phase × contingency × temperature	106	106	1	57.05	0.1460	0.7037628
Phase × contingency × intensity	16	16	1	1091.92	0.0221	0.8819608
Phase × temperature × intensity	979	979	1	1091.92	1.3518	0.2452190
Contingency × temperature × intensity	15,142	15,142	1	1091.92	20.9133	5.354 e‐06***
Phase × contingency × temperature × intensity	310	310	1	1091.92	0.4286	0.5128109

*Note:* Complex model of the un‐/pleasantness ratings in the avoidance task with the factors phase (baseline vs. learning phase), intensity (low vs. high), contingency (contingent vs. noncontingent), temperature (+2°C vs. −2°C).

***p < 0.001, *p < 0.05.

#### 3.1.3 Discrimination Task

Table 8 presents the absolute values of intensity and un‐/pleasantness ratings the participants gave during the discrimination task during the baseline as well as during the learning phase.

**TABLE 8:** Absolute values of the intensity and un‐/pleasantness ratings in the discrimination task.

	Contingent	Noncontingent
*Intensity*		
Baseline	108.26 (29.23)	108.58 (29.19)
Learning phase	109.69 (33.49)	108.06 (33.30)
*Unpleasantness*		
Baseline	0.58 (24.04)	−1.67 (24.30)
Learning phase	−2.75 (27.26)	1.48 (25.28)

*Note:* Mean and standard deviations (in parentheses) of intensity and un‐/pleasantness ratings in the discrimination task in the baseline and the learning phase for the groups with contingent and non‐contingent reinforcement.

Intensity ratings did neither differ significantly between baseline and learning phase (main effect “phase”: *F*_(1)_ = 0.21, *p* = 0.651, $\eta_{p}^{2}$ < 0.01) nor between contingent and noncontingent reinforcement (main effect “contingency”: *F*_(1)_ = 0.009, *p* = 0.925, $\eta_{p}^{2}$ < 0.01; interaction “phase” × “contingency”: *F*_(1)_ = 0.23, *p* = 0.631, $\eta_{p}^{2}$ < 0.01).

Similarly, unpleasantness ratings did neither differ significantly between baseline and learning phase (main effects “phase”: *F*_(1)_ = 0.34, *p* = 0.560, $\eta_{p}^{2}$ < 0.01) nor between contingent and noncontingent reinforcement (main effect “contingency”: *F*_(1)_ < 0.001, *p* = 0.990, $\eta_{p}^{2}$ < 0.01). Although “phase” and “contingency” showed a significant interaction effect (*F*_(1)_ = 4.00, *p* = 0.050, $\eta_{p}^{2}$ = 0.06), but Tukey adjusted *p*‐values did not indicated any significant differences for the post hoc pairwise comparisons.

### 3.2 Thermal Pain Threshold and Tolerance Increased After the Main Experimental Task

Table 9 presents the absolute temperatures of pain thresholds and pain tolerances before and after the avoidance and discrimination task, respectively.

**TABLE 9:** Absolute temperatures of the pain threshold and pain tolerance.

	Avoidance task		Discrimination task	
	Contingent	Noncontingent	Contingent	Noncontingent
*Pain threshold*				
Pre‐measurement	43.73 (2.61)	43.64 (2.88)	43.92 (2.22)	44.07 (2.24)
Post‐measurement	45.21 (2.65)	45.45 (2.25)	46.33 (1.97)	46.63 (2.44)
*Pain tolerance*				
Pre‐measurement	48.32 (1.13)	47.76 (1.56)	47.88 (1.31)	48.60 (0.68)
Post‐measurement	48.90 (0.78)	48.32 (1.17)	48.87 (0.70)	49.07 (0.94)

*Note:* The pre‐ and post‐measurements of pain threshold and pain tolerance in °C during the avoidance task and the discrimination task; standard deviations are given here in parentheses.

#### 3.2.1 Avoidance Task

While thermal pain threshold differed significantly between pre‐ and post‐measurement (main effect “time”: *F*_(1)_ = 29.11, *p* < 0.001, $\eta_{p}^{2}$ = 0.33) being higher in the post‐measurement (post hoc comparison: *p* < 0.001, *d* = −0.99), they did not differ between contingent and noncontingent reinforcement (main effect “contingency”: *F*_(1)_ = 0.02, *p* = 0.896, $\eta_{p}^{2}$ < 0.01; interaction effect “time” × “contingency”: *F*_(1)_ = 0.32, *p* = 0.574, $\eta_{p}^{2}$ < 0.01). Similarly, thermal pain tolerance differed significantly between pre‐ and post‐measurement (main effect “time”: *F*_(1)_ = 14.23, *p* < 0.001, $\eta_{p}^{2}$ = 0.21) being higher in the post‐measurement (post hoc comparison: *p* < 0.001, *d* = −0.71), they did not differ between contingent and noncontingent reinforcement (main effect “contingency”: *F*_(1)_ = 3.94, *p* = 0.052, $\eta_{p}^{2}$ = 0.07; interaction effect “time” × “contingency”: *F*_(1)_ = 0.02, *p* = 0.897, $\eta_{p}^{2}$ < 0.01).

#### 3.2.2 Discrimination Task

While thermal pain threshold differed significantly between pre‐ and post‐measurement (main effect “time”: *F*_(1)_ = 90.25, *p* < 0.001, $\eta_{p}^{2}$ = 0.61) being higher in the post‐measurement (post hoc comparison: *p* < 0.001, *d* = −1.75), they did not differ between contingent and noncontingent reinforcement (main effect “contingency”: *F*_(1)_ = 0.16, *p* = 0.695, $\eta_{p}^{2}$ < 0.01; interaction effect “time” × “contingency”: *F*_(1)_ = 0.05, *p* = 0.823, $\eta_{p}^{2}$ < 0.01). The thermal pain tolerance differed significantly between pre‐ and post‐measurement (main effect “time”: *F*_(1)_ = 31.53, *p* < 0.001, $\eta_{p}^{2}$ = 0.39) being higher in the post‐measurement (post hoc comparison: *p* < 0.001, *d* = −1.09), and between contingent and noncontingent reinforcement (main effect “contingency”: *F*_(1)_ = 4.07, *p* = 0.049, $\eta_{p}^{2}$ = 0.07) being higher with the noncontingent reinforcement (post hoc comparison: *p* = 0.049, *d* = −0.62). There was no significant interaction effect between these two factors (interaction effect “time” × “contingency”: *F*_(1)_ = 2.88, *p* = 0.096, $\eta_{p}^{2}$ = 0.05).

These results suggest pain habituation across the testing session independent of the specific experimental manipulations during the sessions.

### 3.3 Sensation Seeking and Impulsivity Are Associated With Learning of Pain Avoidance and Pain Discrimination

In the avoidance task, the lower impulsivity as a trait (BIS‐15; *r* = 0.36, *t*_(28)_ = 2.06, *p* = 0.049), the more increased reaction times in the learning phase compared to baseline. In the discrimination task, the higher the rest avoidance (NISS; *r* = −0.36, *t*_(28)_ = −2.06, *p* = 0.049), the more decreased reaction times in the learning phase compared to baseline. No other correlations were significant, neither for the outcome variables of the avoidance task nor for the discrimination task.

In addition, we explored whether the contingency groups (avoidance‐contingent reinforcement vs. discrimination‐contingent reinforcement) differed in terms of impulsivity and rest avoidance. An independent *t*‐test comparing the BIS‐15 impulsivity scores between the two groups revealed no significant difference (*t*_(58)_ = −0.52, *p* = 0.61), indicating that impulsivity did not differ significantly between the avoidance and discrimination groups. Similarly, an independent *t*‐test comparing the NISS rest avoidance scores showed no significant difference between the groups (*t*_(58)_ = −0.09, *p* = 0.93), suggesting that levels of rest avoidance were similar across the two groups.

In sum, outcome variables in the avoidance task showed associations with impulsivity personality traits, while outcome variables in the discrimination task showed associations with variables related to rest avoidance. Furthermore, there were no significant differences between the two contingency groups in terms of impulsivity or rest avoidance.

## 4 Discussion

The present study investigated the effects of operant conditioning on surrogate measures of emotional‐motivational and sensory‐discriminative pain components. Contingent monetary reinforcement for pain avoidance enhanced success rates and decreased response times compared to a baseline without reinforcement. No such effects were observed with noncontingent reinforcement. These findings support our hypothesis that emotional‐motivational pain responses can be enhanced by operant conditioning. However, such conditioning could not be observed in the discrimination task. In contrast to our hypothesis, monetary reinforcement for correct discrimination behaviour did not result in increased pain discrimination. This lack of effects contrasts with earlier work by Becker and colleagues using a similar task (Becker et al. 2020). Nevertheless, the present results suggest that operant conditioning can be used to modulate avoidance‐related aspects of pain, while discrimination remained unaffected. This observation is in line with previous results showing that different response channels of pain can be differentially modulated by operant conditioning without necessarily showing comparable effects across these channels (Becker et al. 2008, 2011, 2012; Hölzl et al. 2005). In sum, these results emphasise the role of operant conditioning as a learning mechanism shaping the multidimensional experience of pain.

The divergent findings between tasks are further in line with prior research showing dissociations between pain components in chronic pain (Hashmi et al. 2013; Lethem et al. 1983; Mansour et al. 2014). The Fear Avoidance Model of Chronic Pain, for example, posits that chronic pain may involve an exaggerated emotional‐motivational response without marked changes in sensory‐discriminative pain processing (Lethem et al. 1983). Correspondingly, chronic pain has been described more recently to induce a negative hedonic shift related to augmented negative affect and impaired motivated behaviour (Borsook et al. 2016). Operant learning might be one mechanism explaining such increases in emotional‐motivational pain components without necessary changes in sensory‐discriminative processing. In natural settings, sensory‐discriminative pain responses might be not or less affected by operant learning, because pain carries a high informational value, determining adaptive escape responses. In contrast, effects on emotional‐motivational pain responses might be mediated by anticipatory fear responses to the aversiveness of pain (Seymour 2019; Seymour and Mancini 2020). For example, negative reinforcement of pain‐relieving postures may reinforce maladaptive behaviours over time. With several such pairings, such pain behaviour is displayed with less and less perceived pain triggered by fear of pain. Eventually, this process can contribute to the development and maintenance of chronic pain with even non‐painful percepts or cues triggering pain responses, in line with the Fear Avoidance Model of Chronic Pain that states that pain responses and chronification are mediated by fear of pain and its avoidance (Crombez et al. 2012). Corresponding to such a process, the onset of chronic pain is usually not abrupt, but subtle and ongoing (Borsook et al. 2018).

Based on these considerations, one might expect better learning with higher stimulation intensities in the avoidance task. Although stimulation intensity affected reaction times with faster responses to higher intensities, stimulus intensity did not affect success rates in the avoidance task, suggesting that perceived stimulus intensity and unpleasantness did not modulate pain avoidance response. In contrast, task difficulty, defined by the required response speed, influenced both outcomes in the avoidance task: more difficult trials were associated with slower reaction times and lower success rates. Interestingly, high‐intensity stimuli triggered faster reactions without impairing accuracy, perhaps due to increased informational and threat value, which may heighten attention (Eccleston and Crombez 1999; Legrain et al. 2009; van Vliet et al. 2018). However, increased difficulty not necessarily leads to a higher threat value of the pain, but rather to an unspecific increase in motivation.

Contrary to our hypothesis, ratings of intensity and unpleasantness did not change with reinforcement. We had expected generalisation from reinforced behaviours to self‐reports, but such effects were absent. Our results are consistent with prior work showing discrepancies between behavioural and self‐report measures in pain research (Bouajram et al. 2020; Katz and Melzack 1999; Labus et al. 2003), although some studies do report generalisation of conditioned responses to subjective (Glogan et al. 2021, 2023; Meulders 2020; Vandael et al. 2023). Further, it has been shown that changes in pain avoidance as a consequence of operant conditioning can affect and generalise to self‐report ratings (Meulders 2020). The present design did not target generalisation of responses in the conditioning phase specifically, potentially explaining the lack of such generalisation here.

We aimed to use behavioural measures to avoid biases in self‐report, but these assessments remain indirect. Previous studies implemented self‐reports of perceived pain intensity and un−/pleasantness as indicators of sensory‐discriminative and emotional‐motivational pain components (Becker et al. 2015; Becker, Ceko, et al. 2013; Becker, Gandhi, Chen, and Schweinhardt 2017; Becker, Gandhi, et al. 2013; Talbot et al. 2019). While this is intuitively reasonable and because of its simplicity compelling, such ratings are confounded by various cognitive and social factors, for example social desirability and individual concepts (Kienle and Kiene 1997; Schweiker et al. 2017). Furthermore, such ratings only partially represent multifaceted perception, neglecting aspects people might not be able to describe verbally (Becker et al. 2012). To avoid such influences, we aimed to assess sensory‐discriminative and emotional‐motivational pain components behaviourally here. Nevertheless, behavioural assessments are unavoidably surrogate measures, assessing pain components indirectly. One limitation here is that the assessed avoidance behaviour can be used as an estimator of motivational behaviour while it does not represent emotional aspects directly. However, the present design does not allow us to dissect to what extent the avoidance behaviour was driven by an intrinsic motivation to avoid pain versus a task compliance motive, that is, following the given instruction to react as quickly as possible, thus, limiting the interpretation of the avoidance task as a measure of motivational processes related specifically to pain avoidance.

A potential source of the discrepancy of operant effects between the two tasks lies in their reinforcement structures. In the discrimination task, the thermal stimulus returned to baseline after a response, representing a form of negative reinforcement, because participants could influence the stimulus duration with their behaviour. Although participants were not instructed to respond quickly and the task focused on identifying small temperature changes, the possibility to end the stimulation may have introduced a motivational component in all conditions and phases, possibly explaining why reinforcement did not affect learning outcomes differentially. In contrast, the avoidance task explicitly involved both negative reinforcement (successful avoidance of pain in all phases and conditions) and positive reinforcement (monetary rewards for successful avoidance in the learning phase), potentially enhancing conditioning effects. Taken together, the present results pattern does not allow us to conclude that emotional‐motivational and sensory‐discriminative components were modulated differentially by operant conditioning. It remains unclear whether the absence of effects on pain discrimination reflects methodological limitations or an insensitivity of sensory‐discriminative responses to reinforcement.

Possibly adding to the lack of conditioning effects in the discrimination task, success rates were very high in all conditions of the task, leaving not much room for further increase due to reinforcement and causing a ceiling effect. This ceiling effect likely influenced the reaction times, preventing differentiation between contingent and noncontingent reinforcement. Further, here a smaller stimulation area was used to compare to the previous work (7.1 cm^2^ vs. 12.5 cm^2^; Becker et al. 2020), possibly improving perceptual resolution and contributing to ceiling effects.

During data acquisition a technical issue resulted in the application of different temperatures across participants in the avoidance task (+2°C vs. −2°C for high and low intensities), unintentionally creating two subgroups. While this represents a clear limitation of this experiment, this systematic difference in stimulation had no measurable effect on reaction times or success rates. The relative difference between stimuli, rather than their absolute values, seemed to drive behavioural responses. Still, this variability should be addressed in future studies.

While this study focused on perceptual mechanisms in healthy individuals, the findings have also clinical relevance for understanding chronic pain. The selective modulation of avoidance behaviour by operant conditioning, without corresponding changes in pain perception, suggests that learned motivational responses may play a key role in chronic pain development. In clinical settings, such reinforcement learning processes could contribute to maladaptive patterns of avoidance and fear, even in the absence of heightened nociceptive input. These insights highlight the potential of targeting reinforcement mechanisms—such as through exposure‐based therapy or behavioural interventions—to reshape pain‐related behaviour without necessarily altering nociceptive sensitivity.

To conclude, this study supports the idea that operant learning can shape motivational aspects of pain, while sensory‐discriminative aspects remained unaffected under the present conditions. The lack of effect in the discrimination task does not allow us to conclude on differential effects of operant conditioning on different pain components. Nevertheless, it could be speculated based on the present results that operant learning has stronger effects on motivational‐emotional compared to sensory‐discriminative components of pain, but this hypothesis needs to be followed up in future studies. The present results underscore the multidimensional nature of pain and the role of learning in shaping its expression. Future research should examine whether these patterns generalise to clinical populations and contribute to chronic pain maintenance through maladaptive learning processes.

## Author Contributions

This study was designed by S.B., and S.G. The experiments were performed by M.L.F. The data were analysed by M.L.F., and M.L., and the results were critically examined by all authors. M.L.F. had a primary role in preparing the manuscript, which was edited by S.B. All authors have approved the final version of the manuscript and agree to be accountable for all aspects of the work.

## Conflicts of Interest

The authors declare no conflicts of interest.

## Supporting information


**Appendix S1:** ejp70162‐sup‐0001‐AppendixS1.docx.

## References

[ejp70162-bib-0001] Auvray, M. , E. Myin , and C. Spence . 2010. “The Sensory‐Discriminative and Affective‐Motivational Aspects of Pain.” Neuroscience and Biobehavioral Reviews 34, no. 2: 214–223. 10.1016/j.neubiorev.2008.07.008. 18718486

[ejp70162-bib-0002] Beck, A. T. , R. A. Steer , and G. K. Brown . 1996. Manual for the Beck Depression Inventory‐II. Psychological Corporation.

[ejp70162-bib-0003] Becker, S. , M. Ceko , M. Louis‐Foster , et al. 2013. “Dopamine and Pain Sensitivity: Neither Sulpiride nor Acute Phenylalanine and Tyrosine Depletion Have Effects on Thermal Pain Sensations in Healthy Volunteers.” PLoS One 8, no. 11: e80766. 10.1371/journal.pone.0080766. 24236199 PMC3827462

[ejp70162-bib-0004] Becker, S. , W. Gandhi , Y. J. Chen , and P. Schweinhardt . 2017. “Subjective Utility Moderates Bidirectional Effects of Conflicting Motivations on Pain Perception.” Scientific Reports 7, no. 1: 7790. 10.1038/s41598-017-08454-4. 28798478 PMC5552734

[ejp70162-bib-0005] Becker, S. , W. Gandhi , N. M. Elfassy , and P. Schweinhardt . 2013. “The Role of Dopamine in the Perceptual Modulation of Nociceptive Stimuli by Monetary Wins or Losses.” European Journal of Neuroscience 38, no. 7: 3080–3088. 10.1111/ejn.12303. 23841460

[ejp70162-bib-0006] Becker, S. , W. Gandhi , S. Kwan , A. K. Ahmed , and P. Schweinhardt . 2015. “Doubling Your Payoff: Winning Pain Relief Engages Endogenous Pain Inhibition.” ENeuro 2, no. 4: ENEURO.0029‐15.2015. 10.1523/ENEURO.0029-15.2015. PMC4596013 26464995

[ejp70162-bib-0007] Becker, S. , W. Gandhi , F. Pomares , T. D. Wager , and P. Schweinhardt . 2017. “Orbitofrontal Cortex Mediates Pain Inhibition by Monetary Reward.” Social Cognitive and Affective Neuroscience 12, no. 4: 651–661. 10.1093/scan/nsw173. 28119505 PMC5390724

[ejp70162-bib-0008] Becker, S. , D. Kleinböhl , D. Baus , and R. Hölzl . 2011. “Operant Learning of Perceptual Sensitization and Habituation Is Impaired in Fibromyalgia Patients With and Without Irritable Bowel Syndrome.” Pain 152, no. 6: 1408–1417. 10.1016/j.pain.2011.02.027. 21439728

[ejp70162-bib-0009] Becker, S. , D. Kleinböhl , and R. Hölzl . 2012. “Awareness Is Awareness Is Awareness? Decomposing Different Aspects of Awareness and Their Role in Operant Learning of Pain Sensitivity.” Consciousness and Cognition 21, no. 3: 1073–1084. 10.1016/j.concog.2012.03.008. 22521471

[ejp70162-bib-0010] Becker, S. , D. Kleinböhl , I. Klossika , and R. Hölzl . 2008. “Operant Conditioning of Enhanced Pain Sensitivity by Heat‐Pain Titration.” Pain 140, no. 1: 104–114. 10.1016/j.pain.2008.07.018. 18774227

[ejp70162-bib-0011] Becker, S. , M. Löffler , and B. Seymour . 2020. “Reward Enhances Pain Discrimination in Humans.” Psychological Science 31, no. 9: 1191–1199. 10.1177/0956797620939588. 32818387 PMC7513004

[ejp70162-bib-0012] Borsook, D. , C. Linnman , V. Faria , A. M. Strassman , L. Becerra , and I. Elman . 2016. “Reward Deficiency and Anti‐Reward in Pain Chronification.” Neuroscience and Biobehavioral Reviews 68: 282–297. 10.1016/j.neubiorev.2016.05.033. 27246519

[ejp70162-bib-0013] Borsook, D. , A. M. Youssef , N. Barakat , C. B. Sieberg , and I. Elman . 2018. “Subliminal (Latent) Processing of Pain and Its Evolution to Conscious Awareness.” Neuroscience and Biobehavioral Reviews 88: 1–15. 10.1016/j.neubiorev.2018.02.015. 29476771 PMC5985199

[ejp70162-bib-0014] Bouajram, R. H. , C. M. Sebat , D. Love , E. L. Louie , M. D. Wilson , and J. J. Duby . 2020. “Comparison of Self‐Reported and Behavioral Pain Assessment Tools in Critically Ill Patients.” Journal of Intensive Care Medicine 35, no. 5: 453–460. 10.1177/0885066618757450. 29448873

[ejp70162-bib-0015] Clark, L. , A. J. Lawrence , F. Astley‐Jones , and N. Gray . 2009. “Gambling Near‐Misses Enhance Motivation to Gamble and Recruit Win‐Related Brain Circuitry.” Neuron 61, no. 3: 481–490. 10.1016/j.neuron.2008.12.031. 19217383 PMC2658737

[ejp70162-bib-0016] Crombez, G. , C. Eccleston , S. Van Damme , J. W. S. Vlaeyen , and P. Karoly . 2012. “Fear‐Avoidance Model of Chronic Pain: The Next Generation.” Clinical Journal of Pain 28, no. 6: 475–483. 10.1097/AJP.0b013e3182385392. 22673479

[ejp70162-bib-0017] de Souza, J. B. , E. Grossmann , D. M. N. Perissinotti , et al. 2017. “Prevalence of Chronic Pain, Treatments, Perception, and Interference on Life Activities: Brazilian Population‐Based Survey.” Pain Research and Management 2017: 4643830. 10.1155/2017/4643830. 29081680 PMC5634600

[ejp70162-bib-0018] Dong, G. , X. Lin , H. Zhou , and Q. Lu . 2014. “How the Win‐Lose Balance Situation Affects Subsequent Decision‐Making: Functional Magnetic Resonance Imaging Evidence From a Gambling Task.” Neuroscience 272: 131–140. 10.1016/j.neuroscience.2014.04.058. 24814016

[ejp70162-bib-0019] Eccleston, C. , and G. Crombez . 1999. “Pain Demands Attention: A Cognitive‐Affective Model of the Interruptive Function of Pain.” Psychological Bulletin 125, no. 3: 356–366. 10.1037/0033-2909.125.3.356. 10349356

[ejp70162-bib-0020] Faul, F. , E. Erdfelder , A.‐G. Lang , and A. Buchner . 2007. “G*Power 3: A Flexible Statistical Power Analysis Program for the Social, Behavioral, and Biomedical Sciences.” Behavior Research Methods 39, no. 2: 175–191. 10.3758/bf03193146. 17695343

[ejp70162-bib-0075] Franz, M. , M. R. Lemke , T. Meyer , J. Ulferts , P. Puhl , and R. P. Snaith . 1998. “German version of the Snaith‐Hamilton‐Pleasure‐Scale (SHAPS‐D): Assessing Anhedonia in Schizophrenic and Depressive Patients.” Fortschritte Der Neurologie Psychiatrie 66, no. 9: 407–413. 10.1055/s-2007-995279. 9782420

[ejp70162-bib-0021] Gandhi, W. , S. Becker , and P. Schweinhardt . 2013. “Pain Increases Motivational Drive to Obtain Reward, but Does Not Affect Associated Hedonic Responses: A Behavioural Study in Healthy Volunteers.” European Journal of Pain 17, no. 7: 1093–1103. 10.1002/j.1532-2149.2012.00281.x. 23349058

[ejp70162-bib-0022] Gatchel, R. J. , R. Neblett , N. Kishino , and C. T. Ray . 2016. “Fear‐Avoidance Beliefs and Chronic Pain.” Journal of Orthopaedic and Sports Physical Therapy 46, no. 2: 38–43. 10.2519/jospt.2016.0601. 26828236

[ejp70162-bib-0023] Glogan, E. , P. Liu , and A. Meulders . 2023. “Generalization of Costly Pain‐Related Avoidance Based on Real‐Life Categorical Knowledge.” Psychological Science 34, no. 7: 809–821. 10.1177/09567976231170878. 37254955

[ejp70162-bib-0024] Glogan, E. , K. Vandael , R. Gatzounis , and A. Meulders . 2021. “When Do we Not Face Our Fears? Investigating the Boundary Conditions of Costly Pain‐Related Avoidance Generalization.” Journal of Pain 22, no. 10: 1221–1232. 10.1016/j.jpain.2021.03.149. 33852945

[ejp70162-bib-0025] Goldberg, D. S. , and S. J. McGee . 2011. “Pain as a Global Public Health Priority.” BMC Public Health 11: 770. 10.1186/1471-2458-11-770. 21978149 PMC3201926

[ejp70162-bib-0026] Goli, Z. , A. Asghari , and A. Moradi . 2016. “Effects of Mood Induction on the Pain Responses in Patients With Migraine and the Role of Pain Catastrophizing.” Clinical Psychology & Psychotherapy 23, no. 1: 66–76. 10.1002/cpp.1939. 25523303

[ejp70162-bib-0027] Grimm, J. 2009. “STAI‐Test: State–Trait‐Anxiety Inventory (Deutsche Version).” Methodenforum Der Universität Wien. MF‐Working Paper, 1–4.

[ejp70162-bib-0028] Hashmi, J. A. , M. N. Baliki , L. Huang , et al. 2013. “Shape Shifting Pain: Chronification of Back Pain Shifts Brain Representation From Nociceptive to Emotional Circuits.” Brain 136, no. 9: 2751–2768. 10.1093/brain/awt211. 23983029 PMC3754458

[ejp70162-bib-0029] Herzberg, P. Y. , H. Glaesmer , and J. Hoyer . 2006. “Separating Optimism and Pessimism: A Robust Psychometric Analysis of the Revised Life Orientation Test (LOT‐R).” Psychological Assessment 18, no. 4: 433–438. 10.1037/1040-3590.18.4.433. 17154764

[ejp70162-bib-0030] Hölzl, R. , D. Kleinböhl , and E. Huse . 2005. “Implicit Operant Learning of Pain Sensitization.” Pain 115, no. 1–2: 12–20. 10.1016/j.pain.2005.01.026. 15836965

[ejp70162-bib-0031] Karsdorp, P. A. , S. Ranson , S. Nijst , and J. W. S. Vlaeyen . 2013. “Goals, Mood and Performance Duration on Cognitive Tasks During Experimentally Induced Mechanical Pressure Pain.” Journal of Behavior Therapy and Experimental Psychiatry 44, no. 2: 240–247. 10.1016/j.jbtep.2012.07.009. 23266602

[ejp70162-bib-0032] Katz, J. , and R. Melzack . 1999. “Measurement of Pain.” Surgical Clinics of North America 79, no. 2: 231–252. 10.1016/S0039-6109(05)70381-9. 10352653

[ejp70162-bib-0033] Kienle, G. S. , and H. Kiene . 1997. “The Powerful Placebo Effect: Fact of Fiction?” Journal of Clinical Epidemiology 50, no. 12: 1311–1318. 10.1016/s0895-4356(97)00203-5. 9449934

[ejp70162-bib-0034] Kleinböhl, D. , R. Hölzl , A. Möltner , C. Rommel , C. Weber , and P. M. Osswald . 1999. “Psychophysical Measures of Sensitization to Tonic Heat Discriminate Chronic Pain Patients.” Pain 81, no. 1–2: 35–43. 10.1016/s0304-3959(98)00266-8. 10353491

[ejp70162-bib-0035] Knutson, B. , G. W. Fong , C. M. Adams , J. L. Varner , and D. Hommer . 2001. “Dissociation of Reward Anticipation and Outcome With Event‐Related fMRI.” Neuroreport 12, no. 17: 3683–3687. 10.1097/00001756-200112040-00016. 11726774

[ejp70162-bib-0036] Krohne, H. W. , B. Egloff , C. W. Kohlmann , and A. Tausch . 1996. “Untersuchungen Mit Einer Deutschen Version der “Positive and Negative Affect Schedule” (PANAS).” Diagnostica 42, no. 2: 139–156. 10.1037/t49650-000.

[ejp70162-bib-0069] Kühner, C. B. , C. Bürger , F. Keller , and M. Hautzinger . 2007. “Reliabilität und Validität des revidierten Beck‐Depressionsinventars (BDI‐II).” Der Nervenarzt 78, no. 6: 651–656. 16832698 10.1007/s00115-006-2098-7

[ejp70162-bib-0037] Labus, J. S. , F. J. Keefe , and M. P. Jensen . 2003. “Self‐Reports of Pain Intensity and Direct Observations of Pain Behavior: When Are They Correlated?” Pain 102, no. 1–2: 109–124. 10.1016/s0304-3959(02)00354-8. 12620602

[ejp70162-bib-0038] Legrain, V. , S. Van Damme , C. Eccleston , K. D. Davis , D. A. Seminowicz , and G. Crombez . 2009. “A Neurocognitive Model of Attention to Pain: Behavioral and Neuroimaging Evidence.” Pain 144, no. 3: 230–232. 10.1016/j.pain.2009.03.020. 19376654

[ejp70162-bib-0039] Lethem, J. , P. D. Slade , J. D. G. Troup , and G. Bentley . 1983. “Outline of a Fear‐Avoidance Model of Exaggerated Pain Perception‐I.” Behaviour Research and Therapy 21, no. 4: 401–408. 10.1016/0005-7967(83)90009-8. 6626110

[ejp70162-bib-0040] Mansour, A. R. , M. A. Farmer , M. N. Baliki , and A. Vania Apkarian . 2014. “Chronic Pain: The Role of Learning and Brain Plasticity.” Restorative Neurology and Neuroscience 32, no. 1: 129–139. 10.3233/RNN-139003. 23603439 PMC4922795

[ejp70162-bib-0041] McNeil, D. W. , and A. J. Rainwater . 1998. “Development of the Fear of Pain Questionnaire—III.” Journal of Behavioral Medicine 21, no. 4: 389–410. 10.1023/a:1018782831217. 9789168

[ejp70162-bib-0042] Melzack, R. , and K. L. Casey . 1968. “Sensory, Motivational, and Central Control Determinants of Pain—A New Conceptual Model.” In The Skin Senses, edited by D. R. Kenshalo , 423–439. Charles C Thomas.

[ejp70162-bib-0043] Meulders, A. 2019. “From Fear of Movement‐Related Pain and Avoidance to Chronic Pain Disability: A State‐Of‐The‐Art Review.” Current Opinion in Behavioral Sciences 26: 130–136. 10.1016/j.cobeha.2018.12.007.

[ejp70162-bib-0044] Meulders, A. 2020. “Fear in the Context of Pain: Lessons Learned From 100 Years of Fear Conditioning Research.” Behaviour Research and Therapy 131: 103635. 10.1016/j.brat.2020.103635. 32417719

[ejp70162-bib-0070] Meule, A. , C. Vögele , and A. Kübler . 2001. “Psychometrische Evaluation der deutschen Barratt Impulsiveness Scale–Kurzversion (BIS‐15).” Diagnostica 57, no. 3: 126–133.

[ejp70162-bib-0045] Meyer, K. , H. Sprott , and A. F. Mannion . 2008. “Cross‐Cultural Adaptation, Reliability, and Validity of the German Version of the Pain Catastrophizing Scale.” Journal of Psychosomatic Research 64, no. 5: 469–478. 10.1016/j.jpsychores.2007.12.004. 18440399

[ejp70162-bib-0046] Minami, M. 2019. “The Role of the Bed Nucleus of the Stria Terminalis in Pain‐Induced Aversive Motivation.” Current Opinion in Behavioral Sciences 26: 46–53. 10.1016/j.cobeha.2018.10.003.

[ejp70162-bib-0047] Ossipov, M. H. , G. O. Dussor , and F. Porreca . 2010. “Central Modulation of Pain.” Journal of Clinical Investigation 120, no. 11: 3779–3787. 10.1172/JCI43766. 21041960 PMC2964993

[ejp70162-bib-0048] Pfingsten, M. , B. Kröner‐Herwig , E. Leibing , and U. Kronshage . 2000. “Validation of the German Version of the Fear‐Avoidance Beliefs Questionnaire (FABQ).” European Journal of Pain 4, no. 3: 259–266. 10.1053/eujp.2000.0178. 10985869

[ejp70162-bib-0049] Poole, H. , S. White , C. Blake , P. Murphy , and R. Bramwell . 2009. “Depression in Chronic Pain Patients: Prevalence and Measurement.” Pain Practice 9, no. 3: 173–180. 10.1111/j.1533-2500.2009.00274.x. 19298363

[ejp70162-bib-0071] R Core Team . 2022. “R: A Language and Environment for Statistical Computing.” R Foundation for Statistical Computing, Vienna, Austria. https://www.R‐project.org/.

[ejp70162-bib-0050] Rainville, P. , B. Carrier , R. K. Hofbauer , M. C. Bushnell , and G. H. Duncan . 1999. “Dissociation of Sensory and Affective Dimensions of Pain Using Hypnotic Modulation.” Pain 82, no. 2: 159–171. 10.1016/S0304-3959(99)00048-2. 10467921

[ejp70162-bib-0051] Roth, M. , and P. Hammelstein . 2012. “The Need Inventory of Sensation Seeking (NISS).” European Journal of Psychological Assessment 28, no. 1: 11–18. 10.1027/1015-5759/a000085.

[ejp70162-bib-0052] Rousselet, G. A. , and R. R. Wilcox . 2020. “Reaction Times and Other Skewed Distributions.” Meta‐Psychology 4: 1630. 10.15626/mp.2019.1630.

[ejp70162-bib-0053] Scheier, M. F. , C. S. Carver , and M. W. Bridges . 1994. “Distinguishing Optimism From Neuroticism (And Trait Anxiety, Self‐Mastery, and Self‐Esteem): A Reevaluation of the Life Orientation Test.” Journal of Personality and Social Psychology 67, no. 6: 1063–1078. 10.1037//0022-3514.67.6.1063. 7815302

[ejp70162-bib-0054] Schweiker, M. , X. Fuchs , S. Becker , et al. 2017. “Challenging the Assumptions for Thermal Sensation Scales.” Building Research and Information 45, no. 5: 572–589. 10.1080/09613218.2016.1183185.

[ejp70162-bib-0055] Seymour, B. 2019. “Pain: A Precision Signal for Reinforcement Learning and Control.” Neuron 101, no. 6: 1029–1041. 10.1016/j.neuron.2019.01.055. 30897355

[ejp70162-bib-0056] Seymour, B. , and F. Mancini . 2020. “Hierarchical Models of Pain: Inference, Information‐Seeking, and Adaptive Control.” NeuroImage 222: 117212. 10.1016/j.neuroimage.2020.117212. 32739554

[ejp70162-bib-0057] Snaith, R. P. , M. J. Hamilton , S. Morley , A. Humayan , D. Hargreaves , and P. Trigwell . 1995. “A Scale for the Assessment of Hedonic Tone the Snaith‐Hamilton Pleasure Scale.” British Journal of Psychiatry 167, no. 1: 99–103. 10.1192/bjp.167.1.99. 7551619

[ejp70162-bib-0058] Spielberger, C. D. , R. L. Gorsuch , R. E. Lushene , P. R. Vagg , and G. A. Jacobs . 1983. Manual for the State–Trait Anxiety Inventory (Form Y1‐Y2). Consulting Psychologists Press.

[ejp70162-bib-0059] Spinella, M. 2007. “Normative Data and a Short Form of the Barratt Impulsiveness Scale.” International Journal of Neuroscience 117, no. 3: 359–368. 10.1080/00207450600588881. 17365120

[ejp70162-bib-0060] Sullivan, M. J. L. , S. R. Bishop , and J. Pivik . 1995. “The Pain Catastrophizing Scale: Development and Validation.” Psychological Assessment 7, no. 4: 524–532. 10.1037/1040-3590.7.4.524.

[ejp70162-bib-0061] Talbot, K. , V. J. Madden , S. L. Jones , and G. L. Moseley . 2019. “The Sensory and Affective Components of Pain: Are They Differentially Modifiable Dimensions or Inseparable Aspects of a Unitary Experience? A Systematic Review.” British Journal of Anaesthesia 123, no. 2: e263–e272. 10.1016/j.bja.2019.03.033. 31053232 PMC6676053

[ejp70162-bib-0062] Vachon‐Presseau, E. , M. V. Centeno , W. Ren , et al. 2016. “The Emotional Brain as a Predictor and Amplifier of Chronic Pain.” Journal of Dental Research 95, no. 6: 605–612. 10.1177/0022034516638027. 26965423 PMC4924545

[ejp70162-bib-0063] van Vliet, C. M. , A. Meulders , L. M. G. Vancleef , and J. W. S. Vlaeyen . 2018. “The Opportunity to Avoid Pain May Paradoxically Increase Fear.” Journal of Pain 19, no. 10: 1222–1230. 10.1016/j.jpain.2018.05.003. 29777952

[ejp70162-bib-0064] Vandael, K. , B. Vervliet , M. Peters , and A. Meulders . 2023. “Excessive Generalization of Pain‐Related Avoidance Behavior: Mechanisms, Targets for Intervention, and Future Directions.” Pain 164, no. 11: 2405–2410. 10.1097/j.pain.0000000000002990. 37498749 PMC10578424

[ejp70162-bib-0065] Vlaeyen, J. W. S. , and S. J. Linton . 2000. “Fear‐Avoidance and Its Consequences in Chronic Musculoskeletal Pain: A State of the Art.” Pain 85, no. 3: 317–332. 10.1016/S0304-3959(99)00242-0. 10781906

[ejp70162-bib-0066] Waddell, G. , M. Newton , I. Henderson , D. Somerville , and C. J. Main . 1993. “A Fear‐Avoidance Beliefs Questionnaire (FABQ) and the Role of Fear‐Avoidance Beliefs in Chronic Low Back Pain and Disability.” Pain 52, no. 2: 157–168. 10.1016/0304-3959(93)90127-B. 8455963

[ejp70162-bib-0067] Watson, D. , L. A. Clark , and A. Tellegen . 1988. “Development and Validation of Brief Measures of Positive and Negative Affect: The PANAS Scales.” Journal of Personality and Social Psychology 54, no. 6: 1063–1070. 10.1037//0022-3514.54.6.1063. 3397865

[ejp70162-bib-0068] World Medical Association . 2013. “World Medical Association Declaration of Helsinki: Ethical Principles for Medical Research Involving Human Subjects.” Journal of the American Medical Association 310, no. 20: 2191–2194. 10.1001/jama.2013.281053. 24141714

