# Supplementary material for: Differential Operant Conditioning of Emotional‐Motivational and Sensory‐Discriminative Pain Responses
Source: Eur J Pain. 2025 Nov 19;30(1):e70162. doi: 10.1002/ejp.70162 (PMC12630449; doi:10.1002/ejp.70162)
Supplement: Supplementary file 1 — Appendix S1: ejp70162‐sup‐0001‐AppendixS1.docx. [file EJP-30-0-s001.docx]

*Supplementary material:*

Differential operant conditioning of emotional-motivational and sensory-discriminative pain responses

**Melissa Luna Flury^1,2,3^, Martin Löffler^1,4,5^, Shaili Gour^1,2,3^, Susanne Becker^1,5^**

^1^ Integrative Spinal Research Group, Department of Chiropractic Medicine, Balgrist University Hospital, Zurich University of Zurich, Zurich, Switzerland

^2^ University of Zurich, Zurich, Switzerland

^3^ Neuroscience Center Zurich, University of Zurich and ETH Zurich, Zurich, Switzerland

^4^ Institute of Cognitive and Clinical Neuroscience, Central Institute of Mental Health, Medical Faculty Mannheim, Heidelberg University, Mannheim, Germany

^5^ Clinical Psychology, Department of Experimental Psychology, Heinrich Heine University Düsseldorf, Germany

# Supplementary results

## Section 1: Contingent operant reinforcement decreased reaction times and increased success rates of pain avoidance behavior

As a first step in the analyses, we aimed to characterize the effects of the main factors of interest in the simplest statistical model possible with the main factors only, i.e., the within-subject factor “phase” (baseline vs. learning) and the between-subject factor “contingency” (contingent vs. noncontingent). As hypothesized, contingent operant reinforcement decreased reaction times in the learning phase compared to baseline significantly in the avoidance task (Table 1; η_p_^2^ = 0.13). As indicated by post-hoc pairwise comparisons, reaction times decreased significantly with contingent reinforcement in the learning phase compared to baseline (p < .001, d = 0.38), while no change was observable with noncontingent reinforcement (p = .957, d = 0.04). Within the learning phase, reaction times were significantly faster with contingent compared to noncontingent reinforcement (p = .042, d = -0.43) as hypothesized. As a manipulation check, reaction times showed no difference during baseline between the contingent and the noncontingent reinforcement (p = .955, d = -0.09). These findings suggest successful operant conditioning of reaction times in the avoidance task.

|  | Sum Sq | Mean Sq | NumDF | DenDF | F value | Pr (<F) |  |
| --- | --- | --- | --- | --- | --- | --- | --- |
| phase | 0.0271919 | 0.0271919 | 1 | 58.899 | 13.7514 | 0.0004637 | *** |
| contingency | 0.0053509 | 0.0053509 | 1 | 58.888 | 2.7060 | 0.1052984 |  |
| phase x contingency | 0.0175820 | 0.0175820 | 1 | 58.899 | 8.8915 | 0.0041616 | ** |

**Table 1. Simple model of the reaction times in the avoidance task.** Simple model of the reaction times in the avoidance task with the factors phase (baseline vs. learning phase) and contingency (contingent vs. noncontingent). *** p<.001, ** p<.01.

Similar to the reaction times, contingent reinforcement increased number of successful reactions in the learning phase compared to baseline in the avoidance task (Table 2; OR = 1.13 [95% CI: 0.40 to 0.86]). As indicated by post-hoc pairwise comparisons and as hypothesized, participates were more often successful in the learning phase compared to baseline with contingent reinforcement (p < .001, d = -0.14), but not with noncontingent reinforcement (p = .690, d = -0.03). Despite these effects, number of successful reactions was not significantly different between contingent and noncontingent reinforcement in the learning phase (p = .242, d = 0.09). As for the reaction times and as a manipulation check, number of successful reactions did not differ significantly between contingent and the noncontingent reinforcement during baseline (p = .915, d = -0.03). Overall, the pattern of these findings hints at successful operant conditioning of success rates in the avoidance task, although not all comparisons reached significance.

|  | Chisq | Df | Pr (>Chisq) |  |
| --- | --- | --- | --- | --- |
| (intercept) | 10.9142 | 1 | 0.0009543 | *** |
| phase | 32.0193 | 1 | 1.527e-08 | *** |
| contingency | 0.4238 | 1 | 0.5150389 |  |
| phase x contingency | 10.1574 | 1 | 0.0014372 | ** |

**Table 2. Simple model of the success rates in the avoidance task.** Simple model of the success rates in the avoidance task with the factors phase (baseline vs. learning phase) and contingency (contingent vs. noncontingent). *** p<.001, ** p<.01.

## Section 2: ***Contingent operant reinforcement did not affect reaction times and success rates of discriminative pain behavior differentially compared to noncontingent reinforcement***

Similar to the avoidance task, in the discrimination task, we first used the simplest statistical model possible with the main factors only, i.e., the within-subject factor “phase” (baseline vs. learning) and the between-subject factor “contingency” (contingent vs. noncontingent) to characterize the effects of the main factors of interest. In contrast to the avoidance task, reaction times showed an overall increase from the baseline to the learning phase as indicated by a significant main effect of “phase” (Table 3; η_p_^2^ = 0.67). While reaction times differed between contingent and noncontingent reinforcement with overall faster reaction times with contingent reinforcement (Table 3; η_p_^2^ = 0.08), phase and contingency did not show an interaction effect (Table 3; η_p_^2^ < 0.01). These findings indicate an increase in reaction times in the learning compared to the baseline phase with no differential effects of the contingencies, suggesting that operant conditioning was not successful.

|  | Sum Sq | Mean Sq | NumDF | DenDF | F value | Pr (<F) |  |
| --- | --- | --- | --- | --- | --- | --- | --- |
| phase | 7.8202 | 7.8202 | 1 | 59.114 | 122.5794 | 4.818e-16 | *** |
| contingency | 0.3532 | 0.3532 | 1 | 62.623 | 5.5367 | 0.02178 | * |
| phase x contingency | 0.0084 | 0.0084 | 1 | 59.599 | 0.1318 | 0.71789 |  |

**Table 3. Simple model of the reaction times in the discrimination task.** Simple model of the reaction times in the discrimination task with the factors phase (baseline vs. learning phase) and contingency (contingent vs. noncontingent). *** p<.001, * p<.05.

Number of correct answers in the discrimination task were higher in the learning phase compared to baseline (Table 4; OR = 2.97 [95% CI: 1.06 to 1.47]) with no differences for contingent and noncontingent reinforcement (Table 4; “contingency”: OR = 1.25 [95% CI: 0.92 to 1.55]; “phase” x “contingency”: OR = 1.23 [95% CI: 0.81 to 1.36]). As a note of caution, success rates were in all conditions very high, potentially causing ceiling effects.

|  | Chisq | Df | Pr (>Chisq) |  |
| --- | --- | --- | --- | --- |
| (intercept) | 143.8975 | 1 | <2e-16 | ***** |
| phase | 6.4711 | 1 | 0.01096 | * |
| contingency | 2.5161 | 1 | 0.11269 |  |
| phase x contingency | 0.0792 | 1 | 0.77837 |  |

**Table 4. Simple model of the success rates in the discrimination task.** Simple model of the success rates in the discrimination task with the factors phase (baseline vs. learning phase) and contingency ( contingent vs. noncontingent). *** p<.001, * p<.05.
